# Mechanism of pathogen recognition by human dectin-2

**DOI:** 10.1074/jbc.M117.799080

**Published:** 2017-06-26

**Authors:** Hadar Feinberg, Sabine A. F. Jégouzo, Maximus J. Rex, Kurt Drickamer, William I. Weis, Maureen E. Taylor

**Affiliations:** From the ‡ Departments of Structural Biology and Molecular and Cellular Physiology, Stanford University School of Medicine, Stanford, California 94305 and; the § Department of Life Sciences, Imperial College London, London SW7 2AZ, United Kingdom

**Keywords:** carbohydrate-binding protein, glycobiology, lectin, macrophage, protein structure, CLEC6A

## Abstract

Dectin-2, a C-type lectin on macrophages and other cells of the innate immune system, functions in response to pathogens, particularly fungi. The carbohydrate-recognition domain (CRD) in dectin-2 is linked to a transmembrane sequence that interacts with the common Fc receptor γ subunit to initiate immune signaling. The molecular mechanism by which dectin-2 selectively binds to pathogens has been investigated by characterizing the CRD expressed in a bacterial system. Competition binding studies indicated that the CRD binds to monosaccharides with modest affinity and that affinity was greatly enhanced for mannose-linked α1–2 or α1–4 to a second mannose residue. Glycan array analysis confirmed selective binding of the CRD to glycans that contain Manα1–2Man epitopes. Crystals of the CRD in complex with a mammalian-type high-mannose Man_9_GlcNAc_2_ oligosaccharide exhibited interaction with Manα1–2Man on two different termini of the glycan, with the reducing-end mannose residue ligated to Ca^2+^ in a primary binding site and the nonreducing terminal mannose residue occupying an adjacent secondary site. Comparison of the binding sites in DC-SIGN and langerin, two other pathogen-binding receptors of the innate immune system, revealed why these two binding sites accommodate only terminal Manα1–2Man structures, whereas dectin-2 can bind Manα1–2Man in internal positions in mannans and other polysaccharides. The specificity and geometry of the dectin-2-binding site provide the molecular mechanism for binding of dectin-2 to fungal mannans and also to bacterial lipopolysaccharides, capsular polysaccharides, and lipoarabinomannans that contain the Manα1–2Man disaccharide unit.

## Introduction

Dectin-2 was originally described in mice as a mannose-binding receptor encoded in the cluster of genes for natural killer cell receptors (1). The dectin-2 polypeptide contains an extracellular C-terminal C-type carbohydrate-recognition domain (CRD)^3^ linked to a transmembrane domain and a short N-terminal cytoplasmic domain. Unlike most of the receptors in this gene cluster, dectin-2 is expressed on monocytes and macrophages. Subsequent analysis revealed that, although mouse dectin-2 is particularly highly expressed on tissue macrophages, it is also found on Kupffer cells, Langerhans cells, and some dendritic cells (2). A similar expression pattern has been observed for the human ortholog (3, 4).

Insight into the function of dectin-2 is provided by the demonstration that, although the receptor lacks obvious signaling motifs in the cytoplasmic domain, at the cell surface it is associated with the common Fc receptor γ subunit (FcRγ), which contains an immunotyrosine activation motif that interacts with Syk kinase, initiating signaling that leads to secretion of a range of cytokines (5). An important role of these cytokines is to direct a Th17 response (5–8). Binding of dectin-2 to a range of pathogens and their surface polysaccharides has been demonstrated. Microbial targets include *Candida albicans* and other fungi, bacteria such as *Klebsiella pneumoniae* and *Mycobacterium tuberculosis* (5–7, 9–13), schistosome egg antigen (14), as well as dust mites (15).

Dectin-2 belongs to a group of receptors on immune cells that contain C-type CRDs and associate with the FcRγ subunit. In humans, these receptors include mincle, a macrophage receptor that binds to trehalose dimycolate on mycobacteria, and blood dendritic cell antigen-2 (BDCA-2) that binds galactose-terminated mammalian glycan such as those on IgG. This group of receptors also includes the macrophage C-type lectin (MCL, dectin-3), which has an overall similar organization and can associate with dectin-2 (16), and the dendritic cell inhibitory receptor (DCIR). In humans, there is one form of DCIR, whereas in mice there are three DCIR proteins and a closely related dendritic cell activation receptor (DCAR).

Because dectin-2 plays a key role in the innate immune response to fungi and bacteria, it is important to understand the molecular basis for recognition of these micro-organisms. The work described here demonstrates the importance of a specific disaccharide, Manα1–2Man, in binding to dectin-2 and provides a structural basis to explain how dectin-2 interacts with this motif in the context of surface sugars of potential pathogens.

## Results

### Expression of dectin-2 CRD

**Figure 1.**
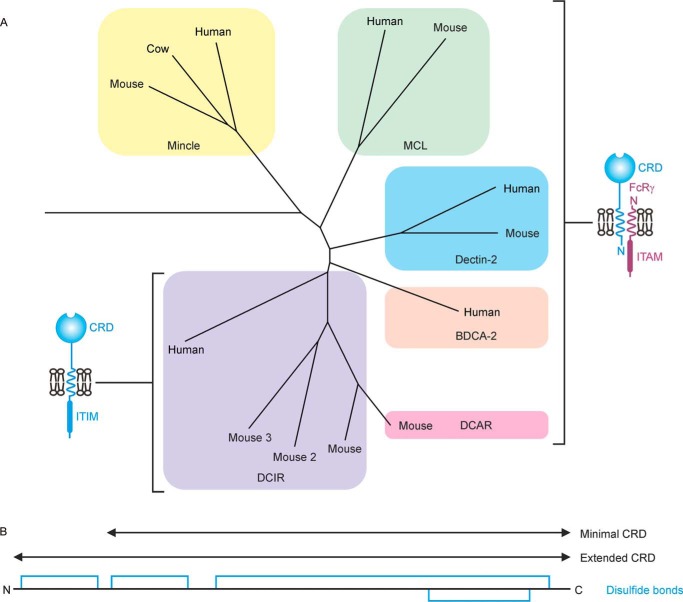
**Organization of human dectin-2 and related proteins containing potential C-type carbohydrate-recognition domains.** *A*, sequence relationships between the CRDs of receptors linked with cell signaling. Sequence similarity with the CRDs is shown in a phylogram, and the organization of the full receptor polypeptides is summarized. *ITAM*, immunotyrosine activation motif; *ITIM*, immunotyrosine inhibitory motif. *B*, arrangement of disulfide bonds in the CRDs.

**Figure 2.**
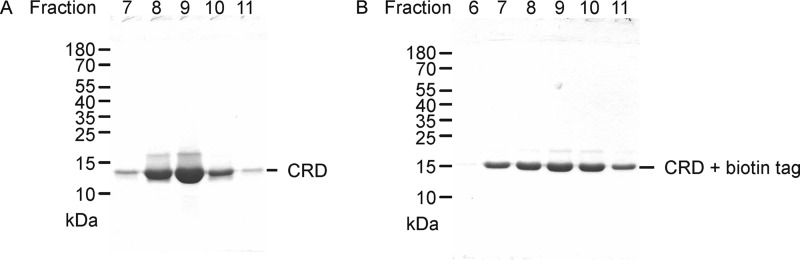
**SDS-PAGE of purified CRD from human dectin-2.** Fractions from the affinity purification of the untagged CRD (*A*) and the biotin-tagged CRD (*B*) were analyzed on 17.5% gels, which were stained with Coomassie Blue.

The sequences of CRDs in receptors that associate with the FcRγ subunit form a closely related cluster (Fig. 1*A*) (17). This cluster also contains DCIR, which does not associate with FcRγ, but contain immunotyrosine inhibitory motifs. One feature shared by the CRDs in all of these families is the presence of an extra disulfide bond compared with most other C-type CRDs, which extends the minimal CRD (Fig. 1*B*). Based on the previously characterized CRDs from mincle and BDCA-2, a region of dectin-2 comprising an extended C-type CRD was defined and cDNAs encoding the extended CRD of dectin-2 were generated, using the polymerase chain reaction, from a human lung cDNA library. Primers were designed for amplification of the untagged CRD and the CRD with a 14-amino acid C-terminal extension that is a target for biotinylation by the enzyme biotin ligase (18). These cDNAs were inserted into the vector pT5T (19) to generate bacterial expression systems. Both forms of the protein were expressed as inclusion bodies in a T7-driven expression system and refolded by renaturation from guanidine-HCl by dialysis in the presence of Ca^2+^. Active protein was purified by affinity chromatography on columns of mannose-Sepharose (Fig. 2). Biotinylation of the C-terminal extension was ensured by co-expression of the biotin ligase gene.

### Binding of dectin-2 to ligands containing Manα1–2Man

**Figure 3.**
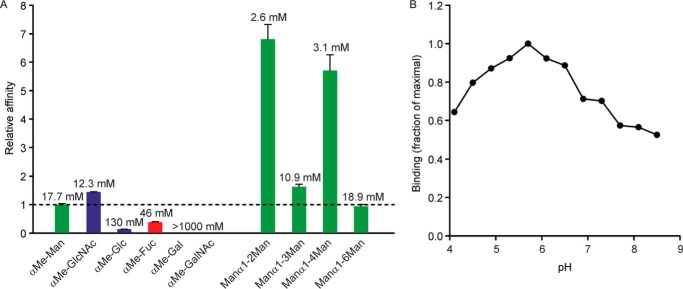
**Solid-phase binding assays for dectin-2 CRD.** *A*, binding competition assays to compare the affinities of dectin-2 for mono- and disaccharide ligands. Inhibition (*K_I_*) constants were determined in the presence of sub-saturating levels of ^125^I-Man-BSA, so that these approximate the inhibitor dissociation constants (59). Relative affinities were calculated by dividing *K_I_* for α-methyl-Man by the *K_I_* values for each of the other sugars. The value at the *top* of each *bar* is the *K_I_* in mm, although the *height* of the *bar* represents the relative *K_I_*. Results shown are means for three separate assays, each conducted in duplicate, with standard deviations shown as *error bars. B*, pH dependence of ^125^I-Man-BSA binding to immobilized CRD from dectin-2. Results have been normalized to the maximal binding, observed at pH 5.7. Data are representative of two assays, each performed in duplicate.

Biotin-tagged CRD from human dectin-2 was immobilized on streptavidin-coated wells and probed with ^125^I-mannose-BSA. By using this reporter ligand at concentrations well below saturation, the affinity for a soluble ligand could be determined in a competition assay that yields an inhibition constant (*K_I_*) that closely approximates the *K_D_* for the competing ligand. Using this format, the binding of various monosaccharides was compared (Fig. 3*A*). The primary interaction of C-type CRDs with their ligands is usually through the 3- and 4-OH groups of a single monosaccharide residue. Although mannose, glucose, and GlcNAc share a common equatorial orientation of these two OH groups, they differ significantly in their binding affinity for dectin-2. A similar spectrum of affinities, in which mannose and GlcNAc bind with similar affinity and glucose binds more weakly, has been observed for several other C-type CRDs, including BDCA-2 (17). As is also typical for CRDs that bind to mannose, fucose is also a ligand, but galactose and GalNAc are not.

Based on the reported interactions of dectin-2 with mannan-type polysaccharides, the interaction with a series of mannose disaccharides was also measured using the competition assay (Fig. 3*A*). The results reveal that disaccharides with α1–2 and α1–4 linkages bind with significantly enhanced affinities (*K_I_* = 2.6 and 3.1 mm, respectively) compared with mannose (*K_I_* = 17.7 mm), suggesting that there is an extended binding site that accommodates a second mannose residue in some of the mannose disaccharides. The solid phase binding assay also provided a means of characterizing the ability of dectin-2 to release ligands in a low pH environment that would be encountered in the endocytic pathway (Fig. 3*B*). Binding is observed across a wide pH range and does not show the rapid decrease below pH 6 that is observed for many recycling endocytic receptors (20). Binding at low pH may allow interaction with pathogens even in relatively low pH environments.

**Figure 4.**
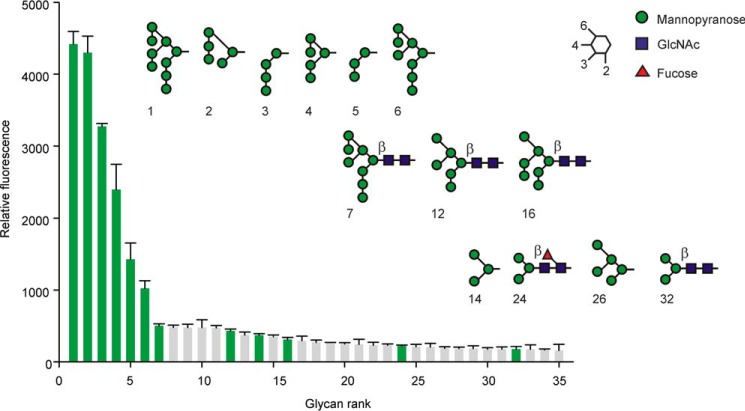
**Glycan array analysis of dectin-2 binding to oligosaccharide ligands.** Glycans giving the strongest signals are shown in rank order, with glycans that contain three or more mannose residues highlighted in *green*. The structures of these glycans are shown. Values shown are the mean signals for six spots of each glycan with standard deviations shown as *error bars*. The mean signal for the remaining 574 glycans on the array was 27. Complete glycan array data are provided in supplemental Table 1.

Further insight into the target ligands for dectin-2 was obtained from glycan array analysis (Fig. 4). An earlier investigation with a more limited array revealed binding of mouse dectin-2 to high-mannose-type mammalian oligosaccharides among the 109 glycans on the array (9). These results were extended by probing a more extensive set of 609 glycans in a micro-array format with the biotin-tagged dectin-2 CRD complexed with fluorescently labeled streptavidin. The top row of structures in the figure highlights six glycans that give the highest signals, at least 2-fold higher than for any other glycan. Each of these oligosaccharides contains one or more Manα1–2Man moieties. Glycans in the bottom row, which contain terminal mannose residues but none in Manα1–2Man linkage, give relatively weak signals. Surprisingly, the three glycans in the middle row, which are analogous to structures in the top row but linked to a chitobiose core, failed to bind as well. Similar results have been noted for other C-type CRDs that interact with high-mannose oligosaccharides (21), which may reflect differences in the efficiency of oligosaccharide immobilization on the array or in the way they are displayed. Nevertheless, combined with the binding competition results, the glycan array data indicate that the Manα1–2Man portion of oligosaccharide ligands is a primary target for dectin-2 binding.

### Structural basis for recognition of Manα1–2Man by dectin-2

**Figure 5.**
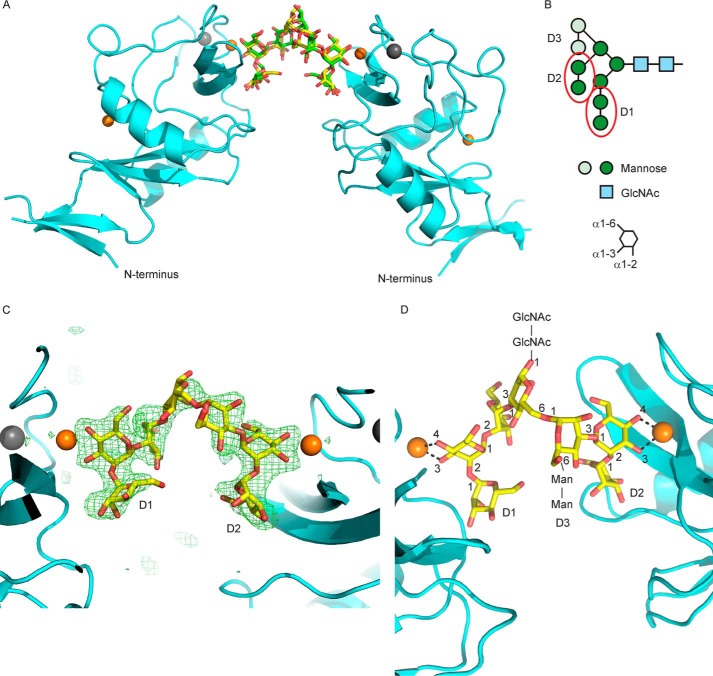
**Arrangement of CRD and oligosaccharide ligand in dectin-2 crystals.** *A*, two asymmetric units are shown, related by a vertical 2-fold axis in the plane of the page. Carbon atoms in the two possible orientations of the oligosaccharide ligand are indicated in *yellow* and *green*. Oxygen atoms are shown in *red*; Ca^2+^ atoms are shown as *orange spheres*, and Na^+^ atoms are shown as a *gray spheres. B*, schematic diagram of the Man_9_GlcNAc_2_ ligand, with the seven mannose residues visible in the crystal shown in *dark green* and the two terminal Manα1–2Man disaccharides visible in the crystal structure circled in *red*. The three branches are numbered D1, D2, and D3. *C*, omit map showing electron density for the sugar ligand contoured at 3σ. *D*, close-up view of the oligosaccharide ligand, with linkages, all in the α-configuration, is indicated. Carbon atoms are in *yellow* and other atoms are as in *A*. Sites of attachment of the four sugar residues not visible in the crystal structure are also indicated. Only one of the two orientations of the oligosaccharide is shown in *C* and *D*.

The structural basis for the selective binding of dectin-2 to ligands containing Manα1–2Man was investigated by analysis of crystals obtained with the untagged form of the CRD in combination with Man_9_GlcNAc_2_ oligosaccharide isolated from soybean agglutinin. The structure was solved by molecular replacement using a model prepared from the coordinates for the CRD of cow mincle (22). The molecular replacement solution revealed that there was one monomer in the asymmetric unit, and difference Fourier maps showed two branches of the Man_9_GlcNAc_2_ carbohydrate cross-linking two protein molecules related by a 2-fold symmetry axis (Fig. 5*A*). The electron density map consequently shows an average of two different branches of Man_9_GlcNAc_2_ in a single dectin-2 molecule. Specifically, the density is strong for seven mannose residues and is fitted well by the Manα1–2Manα1–2Manα1–3(Manα1–2Manα1–3 Manα1–6)Man portion of the oligosaccharide, comprising branches D1 and D2 (Fig. 5, *B* and *C*). The electron density is clear for the first two α1–2-linked mannose residues at the non-reducing end of each of the two branches. The remaining density corresponds to three more mannose residues where the Manα1–3 (Manα1–6)Man moiety can be fitted into the density in both orientations about the crystallographic 2-fold axis. The carbohydrate was therefore modeled as two symmetry-related molecules each at 50% occupancy (Fig. 5*A*).

The overall fold of the dectin-2 polypeptide is closely similar to that observed for other C-type CRDs, particularly those from mincle and BDCA-2. Mincle, BDCA-2, and dectin-2 are particularly similar in the arrangement of the five key amino acid residues that form a conserved Ca^2+^-binding site, which in turn forms the primary sugar-binding site. Many CRDs, including those in mincle and BDCA-2, bind an auxiliary Ca^2+^ near the conserved site. However, although several of the amino acid side chains that form ligands for this accessory Ca^2+^ are present in dectin-2, in the crystals dectin-2 has a Na^+^ at this site. Replacement of the accessory Ca^2+^ with Na^+^ has been observed in some crystal forms of surfactant protein A and mincle (22, 23). A more remote Ca^2+^ is also observed in dectin-2, at a site analogous to a Ca^2+^ in crystals of mincle and some other C-type CRDs, although no analogous Ca^2+^ is observed in BDCA-2 crystals. The N-terminal extension seen in dectin-2 forms a β-hairpin structure like that present in mincle and BDCA-2, although the length of the loop differs, corresponding to different numbers of amino acids present between the two cysteine residues that are disulfide-bonded to each other to close off the loop.

**Figure 6.**
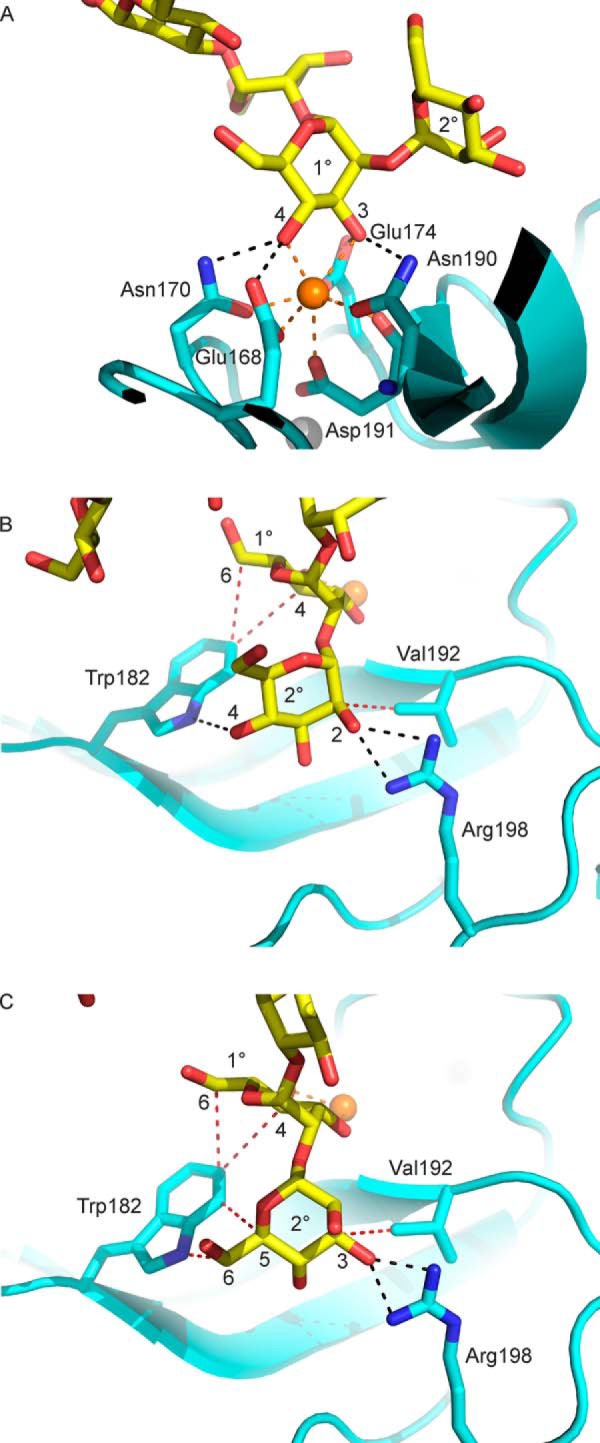
**Details of the sugar-binding sites in the dectin-2 CRD.** *A*, interactions at the primary sugar-binding site, with OH groups 3 and 4 of the sub-terminal mannose residue (*1*°) ligated to the conserved Ca^2+^ through coordination bonds indicated by *orange dotted lines* and hydrogen bonds denoted by *black dotted lines. B* and *C*, interaction of mannose at the secondary binding site for the D1 branch (*B*) and the D2 branch (*C*). Hydrogen bonds between the reducing-end mannose residue (*2*°) and Trp-182 and Arg-198 are shown as *black dotted lines*. van der Waals interactions, shown as *red dotted lines*, are observed between this mannose residue and Val-192 as well as between Trp-182 and the mannose residue in the primary binding site (*1*°). Distances for Ca^2+^ coordination bonds are between 2.4 and 2.5 Å; hydrogen bonds are between 2.4 and 3.4 Å, and van der Waals interactions shown are distances of 3.2 to 4.2 Å.

Because of the distinct covalent structures of the two branches of the oligosaccharide seen in the crystal structure, the model fitted to the electron density provides views of two different terminal trisaccharides bound to dectin-2: Manα1–2Manα1–2Man in the D1 branch and Manα1–2Manα1–3Man in the D2 branch (Fig. 5*C*). In each case, the sub-terminal mannose residue occupies the primary binding site, with 3- and 4-OH groups ligated to the conserved Ca^2+^ and also hydrogen bonded to residues Glu-168, Asn-170, Glu-174, and Asn-190, which in turn are ligated to the Ca^2+^ (shown for the D2 branch in Fig. 6*A*). In addition to this set of interactions, which are common to most C-type CRDs that bind mannose and related sugars, C4 and C6 of mannose in the primary binding site make van der Waals contacts with Trp-182 (Fig. 6, *B* and *C*). These interactions would favor the observed orientation of the mannose residue over the alternative arrangement in which the sugar is rotated 180° by swapping the positions of the 3- and 4-OH groups. Examples of ligands bound in both orientations are observed at roughly equal frequency in other C-type CRDs, and in many cases a CRD can bind mannose in either orientation (24–29). As a result of the orientation being fixed by the interactions with Trp-182 in dectin-2, the mannose residue at the non-reducing terminus occupies a secondary bind site, making hydrogen bonds with Arg-198 as well as contacts with Trp-182 and Val-192.

Binding of the 3- and 4-OH groups to Ca^2+^ in the primary site would preclude binding of Manα1–3 and Manα1–4 disaccharides through the reducing-end mannose residue, although binding of the non-reducing mannose residue would still be possible. For Manα1–3Man and Manα1–6Man disaccharides, the binding competition results suggest that no additional favorable interactions occur with the reducing sugar. In the case of Manα1–3Man, the observed conformation of this disaccharide within the trisaccharide Manα1–2Manα1–3Man, in which the reducing mannose residue is projected away from the surface of the protein, supports this interpretation. Either mannose residue of Manα1–6Man could bind, but the positions of the 1- and 6-OH groups of the mannose residue in the primary binding site suggest that residues linked α1–6 at either side would also project away from the protein. The binding competition results, combined with the structural analysis, suggest that the Manα1–4Man disaccharide binds with the reducing end residue ligated to the conserved Ca^2+^, with binding enhanced by additional interactions with the non-reducing end residue. However, structures containing Manα1–4Man are scarce in biological systems, so it is unclear whether this is a biologically significant interaction.

### Distinct secondary binding sites in related receptors

**Figure 7.**
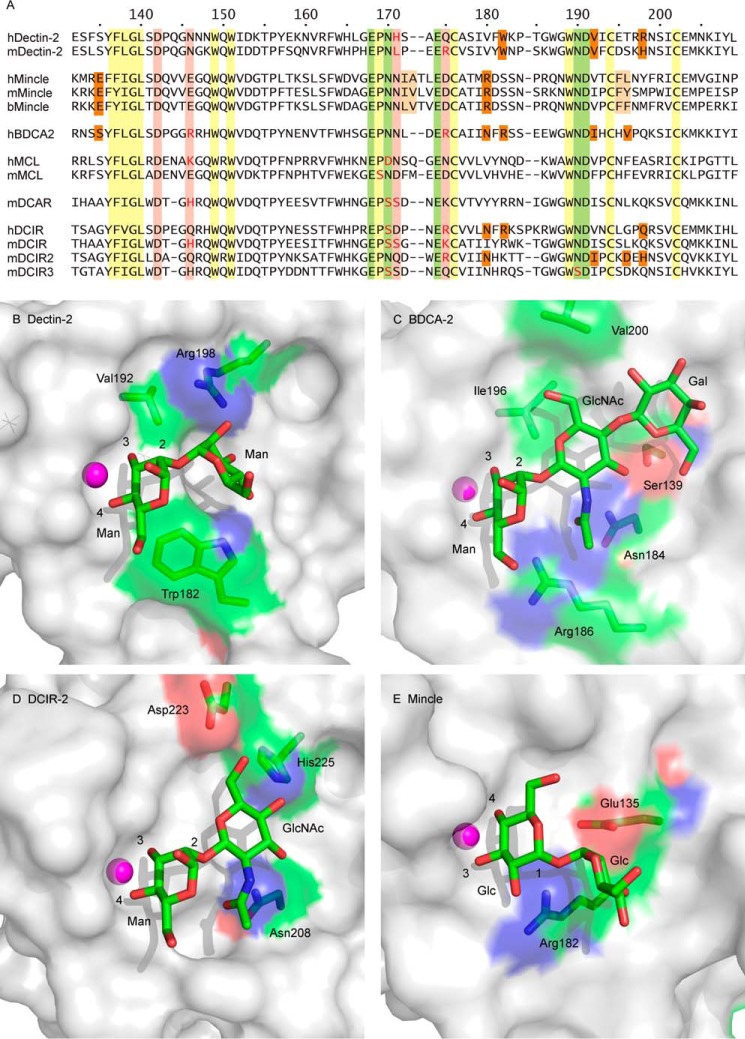
**Comparison of disaccharide-binding sites in signaling sugar-binding receptors.** *A*, sequence comparisons showing C-terminal portions of the CRDs. Residue numbers for human dectin-2 are indicated at the *top*. Conserved amino acid residues, including cysteine residues, that form key parts of the CRD fold are shaded *yellow*; residues that interact with the conserved primary Ca^2+^ are shaded *green*; residues that interact with the adjacent auxiliary Ca^2+^, when it is present, are shaded *pink*; and residues that form the secondary sugar-binding site are shaded *orange*. Residues that do not fit the pattern associated with canonical primary Ca^2+^-binding sites are shown in *red type*. Mannose residues in the primary binding sites of dectin-2 (*B*), BDCA-2 (PDB entry 4ZET) (*C*), and DCIR (PDB entry 3VYK) (*D*) and glucose residue in mincle (PDB entry 4ZRV) (*E*) are shown in approximately the same orientation. *B–E*, carbon atoms are colored *green*; oxygen atoms are colored *red*, and nitrogen atoms are colored *blue*. The glucose residue is rotated 180° compared with the mannose residues, so that the 3- and 4-OH groups have been swapped.

Comparison of the dectin-2 CRD with other relatively closely related proteins in the family of signaling C-type lectins reveals that although many of these domains share a common ability to bind disaccharide ligands, the details of binding differ. Residues that form distinct secondary binding sites in these proteins are mostly clustered in two regions of the sequences (shaded *orange* in Fig. 7*A*). The closest comparison to dectin-2 binding to Manα1–2Man is with BDCA-2 binding to GlcNAcα1–2Man (Fig. 7, *B* and *C*) (17). In each case, the mannose residue in the primary binding site has substituents on the 2-OH group, which enhance the relatively low affinity for mannose monosaccharide through the additional interactions in the secondary binding site. Because this secondary site is adjacent to the axial 2-OH group of mannose, the reducing end sugar in disaccharides containing a sugar with an equatorial 2-OH group in the primary binding site, such as glucose (Fig. 4), would not be correctly positioned to interact with this secondary binding site. Thus, in combination with the secondary binding site, the primary binding site becomes selective for mannose at the reducing end of a disaccharide. Mannose in the secondary binding site in dectin-2 and GlcNAc in the secondary binding site of BDCA-2 interact with distinct types of amino acid side chains (Fig. 7, *B* and *C*). Several of the key interactions in BDCA-2 are with the equatorial 2-acetamido group, which thus serves to distinguish the GlcNAcα1–2Man and Manα1-2Man disaccharides.

Structural evidence suggests that some forms of a third member of the signaling receptor family, DCIR, may bind to GlcNAcα1–2Man in a manner similar to the way that BDCA-2 binds to this disaccharide. In the case of mouse DCIR-2, unpublished glycan array results have been cited as showing specificity for bisected complex *N*-linked glycans, and a crystal structure shows binding to GlcNAcα1–2Man in a larger complex *N*-linked glycan, with the bisecting GlcNAc interacting nearby (30). Interactions with residues analogous to those in BDCA-2 are observed (Fig. 7*D*). A crystal structure of human DCIR has also been determined with bound GlcNAcα1–2Man, but binding to this ligand has not been reported, and different types of assays have produced conflicting evidence about which sugar ligands, if any, human DCIR binds: glycan array analysis reveals binding to sulfated sugars (31); neoglycoprotein binding proximity assay shows binding to mannose- and fucose-containing neoglycoproteins (32); and biotin-polyacrylamide conjugates show binding to Man_3_ and the Lewis^b^ trisaccharide (33). The absence of strong binding even to oligosaccharide ligands may reflect the substitution of serine for one of the asparagine residues that usually forms part of the binding site for the conserved Ca^2+^ (Fig. 7*A*).

Like dectin-2, mincle shows significantly enhanced affinity for a disaccharide ligand compared with monosaccharide, but the only disaccharide ligand that binds with high affinity is trehalose, which is an unusual α1-α1 disaccharide of glucose. In this case, the secondary site accommodates a sugar projected from the 1-OH group rather than the 2-OH group. However, in the α-configuration, this OH group is axial like the 2-OH group of mannose, and the glucose residue in the primary binding site is rotated 180° compared with the mannose residues in dectin-2 and BDCA-2, so the reducing-end sugar projects from the same side of the primary binding site (Fig. 7*E*). Thus, the secondary binding site in mincle is near but somewhat displaced from the secondary sites in dectin-2, BDCA-2, and DCIR.

The remaining member of the signaling receptor group is the macrophage C-type lectin (MCL). Both human and mouse forms for MCL lack one or more of the residues needed to form the canonical primary sugar-binding site, and biochemical analysis of the purified protein failed to demonstrate sugar binding (34). The available NMR structure lacks Ca^2+^, and in a crystal structure without bound sugar ligand, the loop required for binding the auxiliary Ca^2+^ is displaced (35).

### Binding to polysaccharide ligands

**Figure 8.**
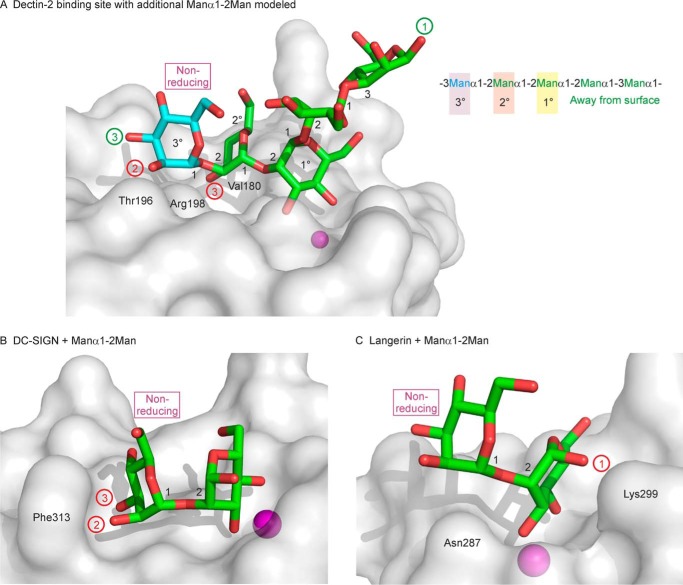
**Binding sites for Manα1–2Man in C-type CRDs.** *A*, Manα1–2Manα1–2Manα1–3 portion of the Man_9_GlcNAc_2_ oligosaccharide bound to dectin-2 is shown, with carbon atoms in *green* and oxygen atoms in *red*. An additional α1–2-linked mannose residue, shown with carbon atoms in *blue* and oxygen atoms in *red,* has been modeled into the structure by superimposing the reducing end mannose residue of the Manα1–2Man disaccharide from PDB entry 2IT6 onto the mannose residue at the non-reducing end of the oligosaccharide ligand. A schematic diagram of the potential binding sites for mannose residues in polysaccharide ligands is also shown. *B*, structure of the Manα1–2Man disaccharide bound to DC-SIGN, from PDB entry 2IT6. *C*, structure of the Manα1–2Man disaccharide bound to langerin, from PDB entry 3P5F. In all three panels, *green numbers* in *circles* denote OH groups that are accessible to further extension, and the addition to OH groups labeled with *red numbers* in *circles* is precluded.

A key feature of the dectin-2-binding site is the ability to accommodate mannose residues that are internal to oligosaccharide chains. The two views provided by the structure show that the central mannose residue in either Manα1–2Manα1–2Man (branch D1) or Manα1–2Manα1–3Man (branch D2) can bind to the primary monosaccharide-binding site, with the reducing end residue in the secondary binding site. Modeling with additional saccharide units demonstrates that the non-reducing end of the trisaccharide can be extended, although there may be some restrictions in the linkages. For example, there is space to model an additional α-linked mannose residue (shown with blue carbon atoms in Fig. 8*A*) attached to the 2-OH group of the residue in the secondary binding site, but addition to the 3-OH group of the secondary site mannose residue is precluded because a mannose residue linked to this group would be superimposed on the side chains of Val-180 and Arg-198. Similarly, a further mannose residue could be added to the 3-OH group of the first modeled mannose residue, but a further mannose residue attached to the 2-OH group would be superimposed directly on the side chain of Thr-196.

The arrangement of the dectin-2-binding site contrasts with other binding sites in C-type lectins that bind mannose residues in the Manα1–2Man disaccharide. For example, DC-SIGN can accommodate the Manα1–2Man disaccharide in an orientation similar to that seen in dectin-2, with the non-reducing mannose residue in a secondary binding site (Fig. 8*B*). However, in DC-SIGN, the 2- and 3-OH groups of the non-reducing mannose residue are directly adjacent to Phe-313, which precludes extension of the disaccharide. A structure of langerin with a Manα1–2Man oligosaccharide has also been described, but the non-reducing mannose residue is projected away from the surface, and the reducing mannose residue is clamped between Asn-287 and Lys-299, so the 1-OH group is inaccessible for attachment of the disaccharide to a larger glycan (Fig. 8*C*).

## Discussion

Binding assays, glycan array analysis, and structural studies have yielded extensive information about the ways that C-type CRDs interact with glycan ligands. Many of the ligand complexes described initially involved sugar residues at the non-reducing termini of glycans, and many of the results observed in glycan array experiments can be explained by interaction of a terminal residue with the primary monosaccharide-binding site (36–38). However, more recent studies have revealed that several C-type CRDs interact with terminal disaccharide or trisaccharide units, in which a sub-terminal sugar residue occupies the primary binding site. Receptors in this group include BDCA-2 and DCIR, whereas other receptors such as DC-SIGN can interact with either terminal or sub-terminal residues in the primary binding site (17, 26, 30). The analysis described here shows a similar arrangement for dectin-2, but it also reveals how this receptor could bind to internal disaccharide units within extended polysaccharides.

**Figure 9.**
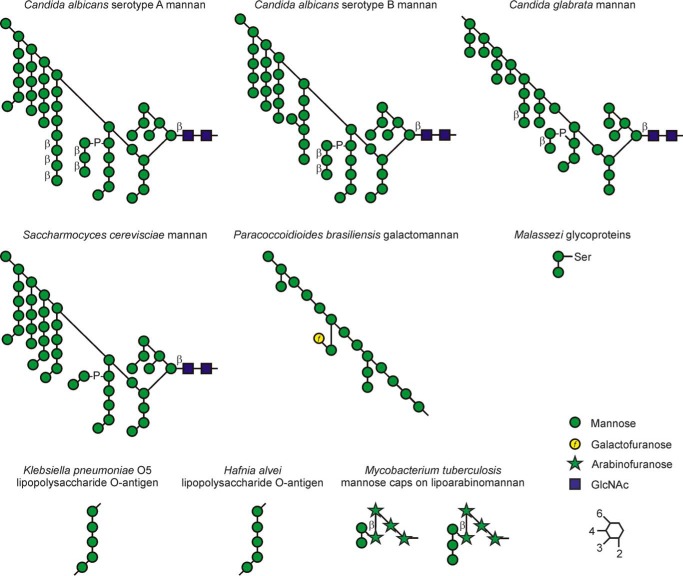
**Structures of microbial ligands for dectin-2.** Linkages are indicated by the *key* at the *bottom right*, with all mannose and arabinose residues in α-configuration and the GlcNAc and galactose linkages in β-configuration except where otherwise indicated. Manα1–2Man motifs are present in each of these ligands, both at the non-reducing termini and at internal positions.

The specificity of the dectin-2-binding site for Manα1–2Man units in polysaccharide ligands can account for most of the observed interaction of mouse and human dectin-2 with a wide range of pathogenic micro-organisms (Fig. 9). For fungi such as *C. albicans*, comparison of different strains and organisms grown under different conditions indicates that α-linked mannans, rather than terminal structures such a β-linked mannose, are responsible for dectin-2 binding (7). The organization of the dectin-2-binding site is consistent with the lack of high affinity binding to β-linked mannose-capping structures. Based on the experimentally observed dihedral angles for β1–2-linked mannans (39), modeling indicates that the reducing-end mannose residue in a Manβ1–2Man disaccharide would be superimposed on the side chain of Arg-198 if the sub-terminal residue is in the primary binding site. The terminal non-reducing mannose residue could occupy the primary binding site, but there would be no secondary interactions to enhance the binding affinity.

Galactomannans on other fungi that bind to dectin-2, such as *Trichophyton* and *Paracoccidioides brasiliensis* (40, 41), also contain α1–6 mannan backbones with α1–2 mannose side chains of various lengths. In contrast, *Cryptococcus neoformans* glucuronoxylomannan, which consists of α1–3-linked mannose and lacks α1–2 branches (42), does not bind. Binding of the pathogenic fungus *Malassezia* is mediated by glycoproteins that contain *O-*linked Manα1–2Man disaccharides (10), whereas bacterial lipopolysaccharides that are targets bear mannose-containing O-antigens with Manα1–2Man in the repeat units (43, 44). Screening an array of mycobacterial glycans confirms that the ligands for dectin-2 are mannose caps such as those found on *Mycobacterium tuberculosis* lipoarabinomannans, which also contain this disaccharide.^4^

The geometry of the binding site, which allows binding of the Manα1–2Man disaccharide in internal positions in a polysaccharide chain, is consistent with the ability of dectin-2 to bind the alternating pattern of α1–2- and α1–3-linked mannose residues in the surface polysaccharides of *K. pneumoniae* and *Hafnia alvei* (43, 44). Although this binding could also be explained by binding to terminal Manα1–2Man units, glycan array results suggest that extensive binding of bacterial polysaccharides is associated with binding to repeat units rather than terminal structures (45), presumably because the former are much more abundant, so there are more potential binding sites and they are at a higher density, which may enhance multivalent binding.

The mode of interaction suggested by this analysis of dectin-2 may be a paradigm for other pathogen-binding receptors. Much of the work to date on the molecular mechanisms of glycan interactions with C-type CRDs, particularly analysis using glycan arrays, has been conducted with mammalian glycans (38). Such data provide important insights into interactions not only with host glycans but also those on viruses and parasites, because these are produced using similar intracellular machinery. However, the results with dectin-2 suggest that further studies with bacterial and fungal glycans will reveal new modes of interaction and in particular novel ways in which more extended binding sites can interact with polysaccharide ligands.

## Experimental procedures

### Analysis of sequences

Gene, cDNA, and protein sequences were obtained from the National Center for Biotechnology Information. The longest predicted isoforms were selected, as these contain the full-length carbohydrate-recognition domains. Phylograms were calculated with Clustal Omega (46) and were rendered with Dendroscope 3 (47).

### Expression constructs

A cDNA encoding the carbohydrate-recognition domain of human dectin-2 (Fig. 1) was amplified from a human lung cDNA library (Clontech) using forward primer aaggatccgatcttggaggatgattaaatggctctcacctgcttcagtgaagggacaaaggtg and reverse primer ctcttctttccaattaagcttctactca, using Advantage2 polymerase (Takara). The forward primer contains a portion of the phage T7 gene 10 followed by a stop codon, an initiator methionine codon, and an alanine codon before the region starting at residue Leu-64 of dectin-2 (19). A biotinylation tag (18) was appended at the C terminus by re-amplification using the initial cDNA as a template with the same forward primer and the extended reverse primer aattcgaagatgagtacggtgagctaaaagacacgaagcttctacagtaagtcaggatccatttagaataagtagag. Amplified fragments were cloned into vector pCRIITopo (Life Technologies, Inc.). After checking of the sequences, BamHI–HindIII fragments were transferred to the pT5T expression vector (19).

### Expression protocol

Inclusion bodies were produced in *Escherichia coli* strain BL21(DE3), in the presence of the pBirA plasmid encoding biotin ligase (18) for the biotin-tagged protein, as described previously for human mincle (48). Inclusion bodies were isolated by sonicating cells from 6 liters of culture in 200 ml of 10 mm Tris-Cl, pH 7.8, four times for 1 min at full power using a Branson 250 sonicator, followed by centrifugation for 15 min at 10,000 × *g* in a Beckman JA-14 rotor. The pellet was dissolved by homogenization in 100 ml of 6 m guanidine HCl, 100 mm Tris-Cl, pH 7.0. The solubilized protein was incubated for 30 min at 4 °C following addition of 2-mercaptoethanol to a final concentration of 0.01%. After centrifugation for 30 min at 40,000 × *g* in a Beckman 70.1Ti rotor, the supernatant was dialyzed against three changes of 2 liters of 0.5 m NaCl, 25 mm Tri-Cl, pH 7.8, 25 mm CaCl_2_ and again centrifuged for 30 min at 100,000 × *g* in the JA-14 rotor. The final supernatant was applied to a 10-ml column of mannose-Sepharose (49), which was washed with 10 ml of 150 mm NaCl, 25 mm Tris-Cl, pH 7.8, 25 mm CaCl_2_ and eluted with 15 aliquots of 1 ml of 150 mm NaCl, 25 mm Tris-Cl, pH 7.8, 2.5 mm EDTA. Fractions containing pure protein were identified by SDS-PAGE on gels containing 17.5% polyacrylamide.

### Binding assays

Biotin-tagged CRD was immobilized on streptavidin-coated wells (Pierce) by incubation for 2 h at 4 °C at a concentration of 20 μg/ml in the presence of 0.1% bovine serum albumin (BSA) in binding buffer consisting of 150 mm NaCl, 25 mm Tris-Cl, pH 7.8, 2.5 mm CaCl_2_. After the wells were washed three times with binding buffer, they were incubated with various concentrations of inhibitors in the presence of 1 μg/ml ^125^I-Man-BSA, prepared by iodination of Man_31_-BSA (E-Y Laboratories) (50), for 2 h at 4 °C, followed by three washes with binding buffer. Methyl glycosides were obtained from Sigma, except methyl GlcNAc and methyl fucose, which were obtained from Carbosynth. Man-Man disaccharides were purchased from Dextra Laboratories. The pH dependence of ligand binding was measured without competing ligand. Incubation with ligand was conducted in buffers containing both 25 mm MES and 25 mm MOPS, at various pH values, in place of Tris.

### Glycan array analysis

A tetrameric form of the CRD was generated by incubating 0.4 mg of biotin-tagged CRD with 0.2 mg of Alexa Fluor 488-labeled streptavidin (Life Technologies, Inc.) overnight at 4 °C. The complex was purified by gel filtration on a Superdex S200 column (GE Healthcare) eluted at 0.5 ml/min with 100 mm NaCl, 10 mm Tris-Cl, pH 7.8, 2.5 mm EDTA. Fractions of 0.5 ml were collected and analyzed for the presence of CRD–streptavidin complex by SDS-PAGE. The complex was used to screen version 5.2 of the mammalian glycan array developed by the Consortium for Functional Glycomics, in which six replicate samples were printed on Nexterion NHS-activated microarray slides (Schott, slide H) with a Piezorray printer from PerkinElmer Life Sciences (51). Binding and washing were conducted in 150 mm NaCl, 20 mm Tris-Cl, pH 7.4, 2 mm CaCl_2_, 2 mm MgCl_2_. Slides were scanned with a ProScanArray instrument from PerkinElmer Life Sciences, and data were processed with the ProScanArray Express Microanalysis System (PerkinElmer Life Sciences). For each set of six replicate spots, the mean and standard deviations were calculated after the highest and lowest values were excluded.

### Crystallization

Man_9_GlcNAc_2_ oligosaccharide was isolated from soybean agglutinin, prepared by affinity chromatography on immobilized GalNAc (Sigma) (52). Hydrazinolysis was performed on 1.5 g of the glycoprotein, followed by re-acetylation and purification of the oligosaccharide by cation-exchange chromatography (53). Further purification was achieved by gel-filtration chromatography on a column of Sephadex G-25 eluted with 1% acetic acid. Crystals of dectin-2 complexed with the oligosaccharide were grown by hanging-drop vapor diffusion at 22 °C using a mixture of 0.9:0.9 μl of protein/reservoir solution in the drop, with the protein solution comprising 2 mg/ml CRD, 5 mm CaCl_2_, 10 mm Tris-Cl, pH 8.0, 25 mm NaCl, and 2 mm Man_9_GlcNAc_2_. The reservoir solution contained 1.0 m NaCl, 6% polyethylene glycol 400, and 0.1 m Tris-Cl, pH 8.0. Crystals were dipped in a freezing solution containing 30% polyethylene glycol 400, 1.0 m NaCl, 0.1 m Tris-Cl, pH 8.0, and 2 mm Man_9_GlcNAc_2_, before being frozen in liquid nitrogen for data collection.

### X-ray diffraction and structure determination

Diffraction data were measured at 100 K on Beamline 12-2 at the Stanford Synchrotron Radiation Laboratory. Diffraction data were integrated with XDS (54) and scaled with AIMLESS (55) to a maximum resolution of 2.4 Å. The statistics are summarized in Table 1. The structure of dectin-2 complexed with Man_9_GlcNAc_2_ was solved by molecular replacement with the program Phaser (56). The model used for molecular replacement was prepared from the coordinates for the CRD of cow mincle (PDB entry 4ZRV), using chain A with three Ca^2+^ ions and no water molecules. The molecular replacement solution confirmed that the space group was C222_1_, with one monomer in the asymmetric unit. The D1 and D2 branches of the oligosaccharide cross-links protein monomers related by a crystallographic 2-fold axis, so the asymmetric unit was modeled with each of these termini bound to the protein at 50% occupancy. Because of the close overlap of the two conformations (Fig. 5*A*), custom non-bonded symmetry exclusions for all seven mannose residues were defined to allow the two conformations to be refined together. Model building and refinement were performed with Coot (57) and PHENIX (58). Refinement included individual positional and isotropic temperature factor refinement. Refinement statistics are shown in Table 1.

**Table 1:** **Crystallographic data collection and refinement statistics** r.m.s.d. indicates root mean square deviation.

Data	Dectin-2 Man_9_GlcNAc_2_ complex
**Data collection statistics**	
Symmetry	C222_1_
Wavelength (Å)	0.97946
Unit cell lengths (Å)	*a* = 64.06, *b* = 78.83, *c* = 76.46
Resolution Å (last shell)	2.4 (2.48–2.4)
*R*_sym_ (last shell)*^a^*	0.080 (0.432)
Mn(I) half-set correlation CC(1/2)	0.99 (0.93)
Mean((*I*)/s(*I*)) (last shell)	12.4 (3.4)
% completeness (last shell)	99.4 (97.3)
No. of unique reflections	7880
Average multiplicity (last shell)	5.2 (5.1)

**Refinement statistics**	
No. of reflections used for refinement	7415
Reflections marked for *R*_free_	391
*R*_free_*^b^*	24.0
*R*_cryst_*^b^*	20.5
Average *B* factor (Å^2^)	45.8
Bond length r.m.s.d. (Å)	0.007
Angle r.m.s.d .(°)	0.53
Ramachandran plot: preferred/allowed/outliers (% in each region) *^c^*	95.0/ 5.0/ 0.0

**PDB code**	5VYB

*^a^ R*_sym_ = Σ*_h_*Σ*_I_*(|*I_i_*(*h*) − 〈*I*(Ih)〉|)/Σ*_h_*Σ*_i_I_i_*(*h*), where *Ii*(*h*) = observed intensity, and 〈*I*(*h*)〉 = mean intensity obtained from multiple measurements.

*^b^ R* and *R*_free_ = 100 × Σ*_h_* |*F_o_*(*h*) − *F_c_*(*h*)|/Σ*_h_F_o_*(*h*), where *F_o_*(*h*) = observed structure factor amplitude and *F_c_*(*h*) = calculated structure factor amplitude for the working and test sets, respectively.

*^c^* Data are as defined in Coot.

## Author contributions

H. F., S. A. F. J., M. J. R., K. D., W. I. W., and M. E. T. conceived the study. S. A. F. J., M. J. R., K. D., and M. E. T. performed the cloning, expression, and binding experiments. H. F. and W. I. W. performed the structural analysis. H. F., K. D., M. E. T., and W. I. W. drafted the manuscript. All authors analyzed the results and approved the final version of the manuscript.

## Supplementary Material

Supplemental Data

## Footnotes

4 R. Zheng, S. A. F. Jégouzo, M. Joe, Y. Bai, H.-A. Tran, K. Shen, J. Saupe, M. E. Taylor, T. L. Lowary, and K. Drickamer, unpublished observations.

## Footnotes

3 The abbreviations used are:

CRD: carbohydrate-recognition domain
PDB: Protein Data Bank
FcRγ: common Fc receptor γ subunit
BSA: bovine serum albumin
BDCA-2: blood dendritic cell antigen 2
MCL: macrophage C-type lectin
DCIR: dendritic cell inhibitory receptor
DCAR: dendritic cell activation receptor.
