# Supplementary material for: Mechanism of pathogen recognition by human dectin-2
Source: J Biol Chem. 2017 Jun 26;292(32):13402–14. doi: 10.1074/jbc.M117.799080 (PMC5555199; doi:10.1074/jbc.M117.799080)
Supplement: Supplemental Data [file supp_292_32_13402__index.html]

Mechanism of pathogen recognition by human dectin-2 — Mechanism of pathogen recognition by human dectin-2 — Mechanism of pathogen recognition by human dectin-2 — Mechanism of pathogen recognition by dectin-2 — Supplemental Data 

# Mechanism of pathogen recognition by human dectin-2

## Supplemental Data

- Supplemental Table 1 (.pdf, 264 KB) - Supplemental Table 1 - Glycan array data
