# Supplementary material for: Mechanism of pathogen recognition by human dectin-2
Source: J Biol Chem. 2017 Jun 26;292(32):13402–14. doi: 10.1074/jbc.M117.799080 (PMC5555199; doi:10.1074/jbc.M117.799080)
Supplement: Supplemental Data [file 10.1074_M117.799080_jbc.M117.799080-1.pdf]

Supplemental Information

Mechanism of Pathogen Recognition by Human Dectin-2

Hadar Feinberg, Sabine A. F. Jégouzo, Maximus J. Rex,
Kurt Drickamer, William I. Weis, and Maureen E. Taylor

Table S1. Screening of Dectin-2 on CFG Glycan Array version 5.2.

SUPPLEMENTAL TABLE 1.

Glycan array results for human dectin-2 binding to the Consortium for Functional Glycomics array version 5.2.

Glycan Numβer	Glycan Structure	Average signal	Standard deviation
316	Manα1-2Manα1-6(Manα1-2Manα1-3)Manα1-6(Manα1-2Manα1-2Manα1-3)Manα-Sp9	4418	178
214	Manα1-2Manα1-2Manα1-6(Manα1-3)Manα-Sp9	4300	231
207	Manα1-2Manα1-2Manα1-3Manα-Sp9	3272	40
208	Manα1-2Manα1-6(Manα1-2Manα1-3)Manα-Sp9	2391	356
209	Manα1-2Manα1-3Manα-Sp9	1427	226
315	Manα1-2Manα1-6(Manα1-3)Manα1-6(Manα1-2Manα1-2Manα1-3)Manα-Sp9	1022	107
211	Manα1-2Manα1-6(Manα1-3)Manα1-6(Manα1-2Manα1-2Manα1-3)Manβ1-4GlcNAcβ1-4GlcNAcβ-Sp12	500	28
571	Galβ1-3GlcNAcβ1-3Galβ1-4GlcNAcβ1-6(Galβ1-3GlcNAcβ1-3Galβ1-4GlcNAβ1-2)Manα1-6(Galβ1-3GlcNAcβ1-3Galβ1-4GlcNAcβ1-2Manα1-3)Manβ1-4GlcNAcβ1-4(Fucα1-6)GlcNAcβ-Sp24	478	31
540	Galβ1-4GlcNAcβ1-3Galβ1-4GlcNAcβ1-2Manα1-6(Galβ1-4GlcNAcβ1-3Galβ1-4GlcNAcβ1-2Manα1-3)Manβ1-4GlcNAcβ1-4GlcNAcβ-Sp12	476	47
21	GlcNAcβ1-6(GlcNAcβ1-4)(GlcNAcβ1-3)GlcNAc-Sp8	475	108
187	GlcNAcβ1-6(GlcNAcβ1-4)GαlNacα-Sp8	472	33
215	Manα1-6(Manα1-3)Manα1-6(Manα1-2Manα1-3)Manβ1-4GlcNAcβ1-4GlcNAcβ-Sp12	429	28
361	Fucα1-2Galβ1-4(Fucα1-3)GlcNAcβ1-2Manα1-6(Fucα1-2Galβ1-4(Fucα1-3)GlcNAcβ1-2Manα1-3)Manβ1-4GlcNAcβ1-4GlcNAβ-Sp20	372	41
213	Manα1-6(Manα1-3)Manα-Sp9	370	20
570	Galβ1-3GlcNAcβ1-3Galβ1-4GlcNAcβ1-3Galβ1-4GlcNAcβ1-6(Galβ1-3GlcNAcβ1-3Galβ1-4GlcNAcβ1-3Galβ1-4GlcNAβ1-2)Manα1-6(Galβ1-3GlcNAcβ1-3Galβ1-4GlcNAcβ1-3Galβ1-4GlcNAcβ1-2Manα1-3)Manβ1-4GlcNAcβ1-4(Fucα1-6)GlcNAcβ-Sp24	349	25
210	Manα1-6(Manα1-2Manα1-3)Manα1-6(Manα1-2Manα1-3)Manβ1-4GlcNAcβ1-4GlcNAcβ-Sp12	308	32
236	Neu5Acα2-3GαlNacα-Sp8	294	62
546	Galβ1-4GlcNAcβ1-3Galβ1-4GlcNAcβ1-3Galβ1-4GlcNAcβ1-2Manα1-6(Galβ1-4GlcNAcβ1-3Galβ1-4GlcNAcβ1-3Galβ1-4GlcNAcβ1-2Manα1-3)Manβ1-4GlcNAcβ1-4GlcNAcβ-Sp12	269	33
606	Neu5Acα2-3Galβ1-4GlcNAcβ1-3Galβ1-4GlcNAcβ1-3Galβ1-4GlcNAcβ1-2Manα1-6(Neu5Acα2-3Galβ1-4GlcNAcβ1-3Galβ1-4GlcNAcβ1-3Galβ1-4GlcNAcβ1-2Manα1-3)Manβ1-4GlcNAcβ1-4GlcNAcβ-Sp12	267	2
364	Fucα1-4(Galβ1-3)GlcNAcβ1-2Manα1-6(Fucα1-4(Galβ1-3)GlcNAcβ1-2Manα1-3)Manβ1-4GlcNAcβ1-4(Fucα1-6)GlcNAcβ-Sp22	250	17
166	Galβ1-4GlcNAcβ1-6(Galβ1-3)GαlNacα-Sp8	242	71
394	Galα1-3Galβ1-3(Fucα1-4)GlcNAcβ1-2Manα1-6(Galα1-3Galβ1-3(Fucα1-4)GlcNAcβ1-2Manα1-3)Manβ1-4GlcNAcβ1-4GlcNAc-Sp19	231	41
544	GlcNAcβ1-3Galβ1-4GlcNAcβ1-3Galβ1-4GlcNAcβ1-2Manα1-6(GlcNAcβ1-3Galβ1-4GlcNAcβ1-3Galβ1-4GlcNAcβ1-2Manα1-3)Manβ1-4GlcNAcβ1-4GlcNAcβ-Sp12	231	19
483	Manα1-6(Manα1-3)Manβ1-4GlcNAcβ1-4(Fucα1-6)GlcNAcβ-Sp19	220	12
605	Neu5Acα2-6Galβ1-4GlcNAcβ1-3Galβ1-4GlcNAcβ1-3Galβ1-4GlcNAcβ1-2Manα1-6(Neu5Acα2-6Galβ1-4GlcNAcβ1-3Galβ1-4GlcNAcβ1-3Galβ1-4GlcNAcβ1-2Manα1-3)Manβ1-4GlcNAcβ1-4GlcNAcβ-Sp12	210	33
314	Manα1-6(Manα1-3)Manα1-6(Manα1-3)Manβ-Sp10	205	52
224	Neu5Acα2-3Galβ1-3GαlNacα-Sp14	187	23
303	GlcNAcβ1-6(Galβ1-4GlcNAcβ1-3)Galβ1-4GlcNAc-Sp0	185	20
222	Fucα1-2(6S)Galβ1-4(6S)Glcβ-Sp0	184	39
350	Manα1-6(Galβ1-4GlcNAcβ1-2Manα1-3)Manβ1-4GlcNAcβ1-4GlcNAcβ-Sp12	177	20
136	Neu5Acα2-6(Galβ1-3)GαlNacα-Sp14	177	29
51	Manα1-6(Manα1-3)Manβ1-4GlcNAcβ1-4GlcNAcβ-Sp13	176	38
235	Neu5Acα2-6(Neu5Acα2-3)GαlNacα-Sp8	175	62
221	Fucα1-2Galβ1-4(6S)GlcNAcβ-Sp8	165	15
81	Fucβ1-3GlcNAcβ-Sp8	160	83
359	Fucα1-2Galβ1-3GlcNAcβ1-2Manα1-6(Fucα1-2Galβ1-3GlcNAcβ1-2Manα1-3)Manβ1-4GlcNAcβ1-4GlcNAcβ-Sp20	149	26
292	Galβ1-4(Fucα1-3)GlcNAcβ1-3Galβ1-3(Fucα1-4)GlcNAcβ-Sp0	142	20
290	Galβ1-4(Fucα1-3)(6S)GlcNAcβ-Sp0	135	44
584	GlcNAcβ1-3Galβ1-4GlcNAcβ1-3Galβ1-4GlcNAcβ1-3Galβ1-4GlcNAcβ1-6(GlcNAcβ1-3Galβ1-4GlcNAcβ1-3Galβ1-4GlcNAcβ1-3Galβ1-4GlcNAβ1-2)Manα1-6(GlcNAcβ1-3Galβ1-4GlcNAcβ1-3Galβ1-4GlcNAcβ1-3Galβ1-4GlcNAcβ1-2Manα1-3)Manβ1-4GlcNAcβ1-4(Fucα1-6)GlcNAcβ-Sp24	133	14
288	Neu5Acα2-3Galβ1-4GlcNAcβ1-6(Galβ1-3)GαlNacα-Sp14	131	62
318	Neu5Acα2-6Galβ1-4GlcNAcβ1-2Manα1-6(Neu5Acα2-3Galβ1-4GlcNAcβ1-2Manα1-3)Manβ1-4GlcNAcβ1-4GlcNAcβ-Sp12	129	18
293	Galβ1-4GlcNAcβ1-3Galβ1-3GlcNAcβ-Sp0	128	30
557	GlcNAcβ1-3Galβ1-4GlcNAcβ1-6(GlcNAcβ1-3Galβ1-4GlcNAcβ1-2)Manα1-6(GlcNAcβ1-3Galβ1-4GlcNAcβ1-2Man a1-3)Manβ1-4GlcNAcβ1-4GlcNAc-Sp24	128	15
523	Galβ1-3GlcNAcβ1-2Manα-Sp0	128	32
262	Fucα1-2Galβ1-4(6S)Glcβ-Sp0	127	27
319	Galβ1-4GlcNAcβ1-2Manα1-6(Neu5Acα2-6Galβ1-4GlcNAcβ1-2Manα1-3)Manβ1-4GlcNAcβ1-4GlcNAcβ-Sp12	124	17
590	Galβ1-4GlcNAcβ1-3Galβ1-4GlcNAcβ1-6(Galβ1-4GlcNAcβ1-3Galβ1-4GlcNAcβ1-3)GαlNacα-Sp14	124	18

586	GlcNAcβ1-3Galβ1-4GlcNAcβ1-3Galβ1-4GlcNAcβ1-3Galβ1-4GlcNAcβ1-3Galβ1-4GlcNAcβ1-6(GlcNAcβ1-3Galβ1-4GlcNAcβ1-3Galβ1-4GlcNAcβ1-3Galβ1-4GlcNAcβ1-3Galβ1-4GlcNAβ1-2)Manα1-6(GlcNAcβ1-3Galβ1-4GlcNAcβ1-3Galβ1-4GlcNAcβ1-3Galβ1-4GlcNAcβ1-3Galβ1-4GlcNAcβ1-2Manα1-3)Manβ	121	8
291	Galβ1-4(Fucα1-3)(6S)Glcβ-Sp0	119	28
371	Galα1-3Galβ1-4(Fucα1-3)GlcNAcβ1-2Manα1-6(Galα1-3Galβ1-4(Fucα1-3)GlcNAcβ1-2Manα1-3)Manβ1-4GlcNAcβ1-4GlcNAcβ-Sp20	115	35
558	Galβ1-4GlcNAcβ1-3Galβ1-4GlcNAcβ1-6(Galβ1-4GlcNAcβ1-3Galβ1-4GlcNAcβ1-2)Manα1-6(Galβ1-4GlcNAcβ1-3Galβ1-4GlcNAcβ1-2Manα1-3)Manα1-4GlcNAcβ1-4GlcNAc-Sp24	114	9
454	Galα1-3(Fucα1-2)Galβ1-3GlcNAcβ1-2Manα1-6(Galα1-3(Fucα1-2)Galβ1-3GlcNAcβ1-2Manα1-3)Manβ1-4GlcNAcβ1-4(Fucα1-6)GlcNAcβ-Sp22	109	13
582	GlcNAcβ1-3Galβ1-4GlcNAcβ1-3Galβ1-4GlcNAcβ1-6(GlcNAcβ1-3Galβ1-4GlcNAcβ1-3Galβ1-4GlcNAβ1-2)Manα1-6(GlcNAcβ1-3Galβ1-4GlcNAcβ1-3Galβ1-4GlcNAcβ1-2Manα1-3)Manβ1-4GlcNAcβ1-4(Fucα1-6)GlcNAcβ-Sp24	109	18
562	GalNAcβ1-4GlcNAcβ1-3GalNAcβ1-4GlcNAcβ-Sp0	108	20
309	GlcNAcβ1-3Man-Sp10	107	19
393	Galα1-3Galβ1-3GlcNAcβ1-2Manα1-6(Galα1-3Galβ1-3GlcNAcβ1-2Manα1-3)Manβ1-4GlcNAcβ1-4GlcNAc-Sp19	105	18
366	Neu5Acα2-6GlcNAcβ1-4GlcNAcβ1-4GlcNAc-Sp21	105	30
374	Fucα1-4(Fucα1-2Galβ1-3)GlcNAcβ1-2Manα1-3(Fucα1-4(Fucα1-2Galβ1-3)GlcNAcβ1-2Manα1-3)Manβ1-4GlcNAcβ1-4GlcNAcβ-Sp19	100	11
604	Neu5Acα2-6Galβ1-4GlcNAcβ1-3Galβ1-4GlcNAcβ1-6(Neu5Acα2-6Galβ1-4GlcNAcβ1-3Galβ1-4GlcNAcβ1-3)GαlNacα-Sp14	99	1
532	Fucα1-4(Galβ1-3)GlcNAcβ1-2 Manα-Sp0	98	10
360	Fucα1-2Galβ1-4GlcNAcβ1-2Manα1-6(Fucα1-2Galβ1-4GlcNAcβ1-2Manα1-3)Manβ1-4GlcNAcβ1-4GlcNAcβ-Sp20	97	24
419	Fucα1-2Galβ1-4GlcNAcβ1-2Manα1-6(Fucα1-2Galβ1-4GlcNAcβ1-2Manα1-3)Manβ1-4GlcNAcβ1-4(Fucα1-6)GlcNAcβ-Sp22	96	14
368	Galβ1-4GlcNAcβ1-2Manα1-6(Galβ1-4GlcNAcβ1-4(Galβ1-4GlcNAcβ1-2)Manα1-3)Manβ1-4GlcNAcβ1-4GlcNAc-Sp21	96	17
475	Galβ1-3GlcNAcβ1-2Manα1-6(GlcNAcβ1-4)(Galβ1-3GlcNAcβ1-2Manα1-3)Manβ1-4GlcNAcβ1-4GlcNAcβ-Sp21	95	10
473	Neu5Acα2-3Galβ1-3GlcNAcβ1-6(Neu5Acα2-3Galβ1-3GlcNAcβ1-2)Manα1-6(Neu5Acα2-3Galβ1-3GlcNAcβ1-2Manα1-3)Manβ1-4GlcNAcβ1-4GlcNAcβ-Sp19	91	21
445	Fucα1-2Galβ1-4(Fucα1-3)GlcNAcβ1-2Manα1-6(Fucα1-2Galβ1-4(Fucα1-3)GlcNAcβ1-4(Fucα1-2Galβ1-4(Fucα1-3)GlcNAcβ1-2)Manα1-3)Manβ1-4GlcNAcβ1-4GlcNAcβ-Sp12	83	16
575	GlcNAcβ1-3Galβ1-4GlcNAcβ1-3Galβ1-4GlcNAcβ1-2Manα1-6(GlcNAcβ1-3Galβ1-4GlcNAcβ1-3Galβ1-4GlcNAcβ1-2Manα1-3)Manβ1-4GlcNAcβ1-4(Fucα1-6)GlcNAcβ-Sp24	83	12
202	GlcAβ-Sp8	81	8
567	Galβ1-4GlcNAcβ1-3Galβ1-4GlcNAcβ1-3Galβ1-4GlcNAcβ1-3Galβ1-4GlcNAcβ1-3Galβ1-4GlcNAcβ1-3Galβ1-4GlcNAcβ1-2Manα1-6(Galβ1-4GlcNAcβ1-3Galβ1-4GlcNAcβ1-3Galβ1-4GlcNAcβ1-3Galβ1-4GlcNAcβ1-3Galβ1-4GlcNAcβ1-3Galβ1-4GlcNAcβ1-2Manα1-3)Manβ1-4GlcNAcβ1-4GlcNAcβ-Sp25	80	11
444	Fucα1-2Galβ1-4 GlcNAcβ1-2Manα1-6(Fucα1-2Galβ1-4GlcNAcβ1-2(Fucα1-2Galβ1-4GlcNAcβ1-4)Manα1-3)Manβ1-4GlcNAcβ1-4GlcNAcβ-Sp12	80	10
589	Galβ1-4GlcNAcβ1-3Galβ1-4GlcNAcβ1-6(Galβ1-3)GαlNacα-Sp14	80	11
602	Neu5Acα2-3Galβ1-4GlcNAcβ1-3Galβ1-4GlcNAcβ1-2Manα1-6(Neu5Acα2-3Galβ1-4GlcNAcβ1-3Galβ1-4GlcNAcβ1-2Manα1-3)Manβ1-4GlcNAcβ1-4GlcNAcβ-Sp12	80	7
426	Galα1-3(Fucα1-2)Galβ1-4GlcNAcβ1-2Manα1-6(Galα1-3(Fucα1-2)Galβ1-4GlcNAcβ1-2Manα1-3)Manβ1-4GlcNAcβ1-4(Fucα1-6)GlcNAcβ-Sp22	79	7
456	GαlNacα1-3(Fucα1-2)Galβ1-3GlcNAcβ1-2Manα1-6(GαlNacα1-3(Fucα1-2)Galβ1-3GlcNAcβ1-2Manα1-3)Manβ1-4GlcNAcβ1-4(Fucα1-6)GlcNAcβ-Sp22	77	13
577	GlcNAcβ1-3Galβ1-4GlcNAcβ1-3Galβ1-4GlcNAcβ1-3Galβ1-4GlcNAcβ1-2Manα1-6(GlcNAcβ1-3Galβ1-4GlcNAcβ1-3Galβ1-4GlcNAcβ1-3Galβ1-4GlcNAcβ1-2Manα1-3)Manβ1-4GlcNAcβ1-4(Fucα1-6)GlcNAcβ-Sp24	77	6
453	GαlNacα1-3(Fucα1-2)Galβ1-4GlcNAcβ1-2Manα1-6(GαlNacα1-3(Fucα1-2)Galβ1-4GlcNAcβ1-2Manα1-3)Manβ1-4GlcNAcβ1-4(Fucα1-6)GlcNAcβ-Sp22	74	17
323	Galβ1-3GlcNAcβ1-2Manα1-6(Galβ1-3GlcNAcβ1-2Manα1-3)Manβ1-4GlcNAcβ1-4GlcNAcβ-Sp19	74	8
550	Galβ1-3GlcNAcβ1-3Galβ1-4GlcNAcβ1-2Manα1-6(Galβ1-3GlcNAcβ1-3Galβ1-4GlcNAcβ1-2Manα1-3)Manβ1-4GlcNAcβ1-4GlcNAc-Sp25	72	9
327	Neu5,9Ac2a2-3Galβ1-4GlcNAcβ-Sp0	71	3
472	Fucα1-2Galβ1-3(Fucα1-4)GlcNAcβ1-2Manα1-6(Fucα1-2Galβ1-3(Fucα1-4)GlcNAcβ1-2Manα1-3)Manβ1-4GlcNAcβ1-4(Fucα1-6)GlcNAcβ1-4(Fucα1-6)GlcNAcβ-Sp19	70	11
28	(3S)Galβ1-3(Fucα1-4)GlcNAcβ-Sp8	68	36
573	GlcNAcβ1-3Galβ1-4GlcNAcβ1-2Manα1-6(GlcNAcβ1-3Galβ1-4GlcNAcβ1-2Manα1-3)Manβ1-4GlcNAcβ1-4(Fucα1-6)GlcNAcβ-Sp24	67	5
583	Galβ1-4GlcNAcβ1-3Galβ1-4GlcNAcβ1-3Galβ1-4GlcNAcβ1-6(Galβ1-4GlcNAcβ1-3Galβ1-4GlcNAcβ1-3Galβ1-4GlcNAβ1-2)Manα1-6(Galβ1-4GlcNAcβ1-3Galβ1-4GlcNAcβ1-3Galβ1-4GlcNAcβ1-2Manα1-3)Manβ1-4GlcNAcβ1-4(Fucα1-6)GlcNAcβ-Sp24	67	7
18	GlcN(Gc)β-Sp8	66	39
351	GlcNAcβ1-2Manα1-6(GlcNAcβ1-2Manα1-3)Manβ1-4GlcNAcβ1-4(Fucα1-6)GlcNAcβ-Sp22	65	29
471	Fucα1-2Galβ1-4(Fucα1-3)GlcNAcβ1-2Manα1-6(Fucα1-2Galβ1-4(Fucα1-3)GlcNAcβ1-2Manα1-3)Manβ1-4GlcNAcβ1-4(Fucα1-6)GlcNAcβ-Sp24	65	10
474	GlcNAcβ1-6(GlcNAcβ1-2)Manα1-6(GlcNAcβ1-2Manα1-3)Manβ1-4GlcNAcβ1-4(Fucα1-6)GlcNAcβ-Sp24	65	24
588	Galβ1-4GlcNAcβ1-3Galβ1-4GlcNAcβ1-3GαlNacα-Sp14	64	5
543	Fucα1-2Galβ1-4GlcNAcβ1-3Galβ1-4GlcNAcβ1-2Manα1-6(Fucα1-2Galβ1-4GlcNAcβ1-3Galβ1-4GlcNAcβ1-2Manα1-3)Manβ1-4GlcNAcβ1-4GlcNAcβ-Sp24	62	16
403	Galα1-4Galβ1-4GlcNAcβ1-2Manα1-6(Galα1-4Galβ1-4GlcNAcβ1-2Manα1-3)Manβ1-4GlcNAcβ1-4GlcNAcβ-Sp24	62	11
545	GlcNAcβ1-3Galβ1-4GlcNAcβ1-3Galβ1-4GlcNAcβ1-2Manα1-6(GlcNAcβ1-3Galβ1-4GlcNAcβ1-3Galβ1-4GlcNAcβ1-2Manα1-3)Manβ1-4GlcNAcβ1-4GlcNAcβ-Sp25	60	10
477	Neu5Acα2-3Galβ1-4GlcNAcβ1-2Manα-Sp0	59	3
24	(3S)Galβ1-4(Fucα1-3)(6S)Glc-Sp0	59	13
195	Glca1-4Glcβ-Sp8	58	9
581	Galβ1-4GlcNAcβ1-3Galβ1-4GlcNAcβ1-6(Galβ1-4GlcNAcβ1-3Galβ1-4GlcNAβ1-2)Manα1-6(Galβ1-4GlcNAcβ1-3Galβ1-4GlcNAcβ1-2Manα1-3)Manβ1-4GlcNAcβ1-4(Fucα1-6)GlcNAcβ-Sp24	58	11
212	Manα1-2Manα1-6(Manα1-2Manα1-3)Manα1-6(Manα1-2Manα1-2Manα1-3)Manβ1-4GlcNAcβ1-4GlcNAcβ-Sp12	57	17

427	Galβ1-3GlcNAcβ1-6(Galβ1-3GlcNAcβ1-2)Manα1-6(Galβ1-3GlcNAcβ1-2Manα1-3)Manβ1-4GlcNAcβ1-4GlcNAcβ-Sp19	57	3
6	Fucα-Sp8	56	16
563	GlcNAcβ1-3Galβ1-4GlcNAcβ1-3Galβ1-4GlcNAcβ1-3Galβ1-4GlcNAcβ1-3Galβ1-4GlcNAcβ1-2Manα1-6(GlcNAcβ1-3Galβ1-4GlcNAcβ1-3Galβ1-4GlcNAcβ1-3Galβ1-4GlcNAcβ1-3Galβ1-4GlcNAcβ1-2Manα1-3)Manβ1-4GlcNAcβ1-4GlcNAcβ-Sp25	56	11
50	Manα1-6(Manα1-3)Manβ1-4GlcNAcβ1-4GlcNAcβ-Sp12	55	9
280	Neu5Gca2-3Galβ1-3(Fucα1-4)GlcNAcβ-Sp0	55	17
2	Glca-Sp8	54	18
391	GalNAcβ1-4(Neu5Acα2-3)Galβ1-4GlcNAcβ1-3GαlNacα-Sp14	53	4
548	GlcNAcβ1-3Galβ1-4GlcNAcβ1-3Galβ1-4GlcNAcβ1-3Galβ1-4GlcNAcβ1-2Manα1-6(GlcNAcβ1-3Galβ1-4GlcNAcβ1-3Galβ1-4GlcNAcβ1-3Galβ1-4GlcNAcβ1-2Manα1-3)Manβ1-4GlcNAcβ1-4GlcNAcβ-Sp25	53	5
410	Galβ1-3GalNAcβ1-4(Neu5Acα2-8Neu5Acα2-3)Galβ1-4Glcβ-Sp0	52	23
585	Galβ1-4GlcNAcβ1-3Galβ1-4GlcNAcβ1-3Galβ1-4GlcNAcβ1-3Galβ1-4GlcNAcβ1-6(Galβ1-4GlcNAcβ1-3Galβ1-4GlcNAcβ1-3Galβ1-4GlcNAcβ1-3Galβ1-4GlcNAβ1-2)Manα1-6(Galβ1-4GlcNAcβ1-3Galβ1-4GlcNAcβ1-3Galβ1-4GlcNAcβ1-3Galβ1-4GlcNAcβ1-2Manα1-3)Manβ1-4GlcNAcβ1-4(Fucα1-6)GlcNAcβ-	52	2
500	Fucα1-2(6S)Galβ1-3(6S)GlcNAcβ-Sp0	51	7
367	Galβ1-4(Fucα1-3)GlcNAcβ1-6(Fucα1-2Galβ1-4GlcNAcβ1-3)Galβ1-4Glc-Sp21	51	5
587	Galβ1-4GlcNAcβ1-3Galβ1-4GlcNAcβ1-3Galβ1-4GlcNAcβ1-3Galβ1-4GlcNAcβ1-3Galβ1-4GlcNAcβ1-6(Galβ1-4GlcNAcβ1-3Galβ1-4GlcNAcβ1-3Galβ1-4GlcNAcβ1-3Galβ1-4GlcNAcβ1-3Galβ1-4GlcNAβ1-2)Manα1-6(Galβ1-4GlcNAcβ1-3Galβ1-4GlcNAcβ1-3Galβ1-4GlcNAcβ1-3Galβ1-4GlcNAcβ1-3Galβ1-4G	51	8
102	Galα1-3(Fucα1-2)Galβ1-3GlcNAcβ-Sp8	50	13
244	Neu5Acα2-6(Neu5Acα2-3Galβ1-3)GαlNacα-Sp14	50	19
200	G-ol-Sp8	49	14
217	Manβ1-4GlcNAcβ-Sp0	49	11
564	Galβ1-4GlcNAcβ1-3Galβ1-4GlcNAcβ1-3Galβ1-4GlcNAcβ1-3Galβ1-4GlcNAcβ1-3Galβ1-4GlcNAcβ1-2Manα1-6(Galβ1-4GlcNAcβ1-3Galβ1-4GlcNAcβ1-3Galβ1-4GlcNAcβ1-3Galβ1-4GlcNAcβ1-3Galβ1-4GlcNAcβ1-2Manα1-3)Manβ1-4GlcNAcβ1-4GlcNAcβ-Sp25	48	5
345	Manα1-6(Neu5Acα2-6Galβ1-4GlcNAcβ1-2Manα1-3)Manβ1-4GlcNAcβ1-4GlcNAc-Sp12	48	6
347	Neu5Acα2-6Galβ1-4GlcNAcβ1-2Manα1-3Manβ1-4GlcNAcβ1-4GlcNAc-Sp12	48	15
1	Galα-Sp8	47	27
198	Glcβ1-4Glcβ-Sp8	47	61
282	Neu5Gca2-3Galβ1-4(Fucα1-3)GlcNAcβ-Sp0	46	19
539	GlcNAcβ1-3Galβ1-4GlcNAcβ1-2Manα1-6(GlcNAcβ1-3Galβ1-4GlcNAcβ1-2Manα1-3)Manβ1-4GlcNAcβ1-4GlcNAcβ-Sp25	46	3
353	Galβ1-3GlcNAcβ1-2Manα1-6(Galβ1-3GlcNAcβ1-2Manα1-3)Manβ1-4GlcNAcβ1-4(Fucα1-6)GlcNAcβ-Sp22	46	15
240	Neu5Acα2-3Galβ1-3(Fucα1-4)GlcNAcβ1-3Galβ1-4(Fucα1-3)GlcNAcβ-Sp0	46	14
7	Fucα-Sp9	45	11
199	Glcβ1-6Glcβ-Sp8	45	23
158	Galβ1-4GalNAcβ1-3(Fucα1-2)Galβ1-4GlcNAcβ-Sp8	44	25
395	Neu5Acα2-3Galβ1-3GlcNAcβ1-2Manα1-6(Neu5Acα2-3Galβ1-3GlcNAcβ1-2Manα1-3)Manβ1-4GlcNAcβ1-4GlcNAc-Sp19	44	29
369	GαlNacα1-3(Fucα1-2)Galβ1-4GlcNAcβ1-2Manα1-6(GαlNacα1-3(Fucα1-2)Galβ1-4GlcNAcβ1-2Manα1-3)Manβ1-4GlcNAcβ1-4GlcNAcβ-Sp20	44	12
580	Galβ1-4GlcNAcβ1-3Galβ1-4GlcNAcβ1-3Galβ1-4GlcNAcβ1-3Galβ1-4GlcNAcβ1-3Galβ1-4GlcNAcβ1-2Manα1-6(Galβ1-4GlcNAcβ1-3Galβ1-4GlcNAcβ1-3Galβ1-4GlcNAcβ1-3Galβ1-4GlcNAcβ1-3Galβ1-4GlcNAcβ1-2Manα1-3)Manβ1-4GlcNAcβ1-4(Fucα1-6)GlcNAcβ-Sp19	44	2
194	GlcNAcβ1-6Galβ1-4GlcNAcβ-Sp8	44	22
201	GlcAa-Sp8	44	29
83	GαlNacα1-3(Fucα1-2)Galβ1-4(Fucα1-3)GlcNAcβ-Sp0	43	7
579	GlcNAcβ1-3Galβ1-4GlcNAcβ1-3Galβ1-4GlcNAcβ1-3Galβ1-4GlcNAcβ1-3Galβ1-4GlcNAcβ1-2Manα1-6(GlcNAcβ1-3Galβ1-4GlcNAcβ1-3Galβ1-4GlcNAcβ1-3Galβ1-4GlcNAcβ1-3Galβ1-4GlcNAcβ1-2Manα1-3)Manβ1-4GlcNAcβ1-4(Fucα1-6)GlcNAcβ-Sp19	43	3
541	Galβ1-4GlcNAcβ1-3Galβ1-4GlcNAcβ1-2Manα1-6(Galβ1-4GlcNAcβ1-3Galβ1-4GlcNAcβ1-2Manα1-3)Manβ1-4GlcNAcβ1-4GlcNAcβ-Sp24	43	3
149	Galβ1-3GlcNAcβ-Sp0	42	35
415	Fucα1-2Galβ1-4(Fucα1-3)GlcNAcβ1-3GαlNacα-Sp14	42	6
576	Galβ1-4GlcNAcβ1-3Galβ1-4GlcNAcβ1-3Galβ1-4GlcNAcβ1-2Manα1-6(Galβ1-4GlcNAcβ1-3Galβ1-4GlcNAcβ1-3Galβ1-4GlcNAcβ1-2Manα1-3)Manβ1-4GlcNAcβ1-4(Fucα1-6)GlcNAcβ-Sp24	41	2
14	Manβ-Sp8	41	12
549	Galβ1-4GlcNAcβ1-3Galβ1-4GlcNAcβ1-3Galβ1-4GlcNAcβ1-3Galβ1-4GlcNAcβ1-2Manα1-6(Galβ1-4GlcNAcβ1-3Galβ1-4GlcNAcβ1-3Galβ1-4GlcNAcβ1-3Galβ1-4GlcNAcβ1-2Manα1-3)Manβ1-4GlcNAcβ1-4GlcNAcβ-Sp25	41	1
447	Galβ1-4GlcNAcβ1-2Manα-Sp0	41	5
12	Galβ-Sp8	40	12
153	Galβ1-4(Fucα1-3)GlcNAcβ1-3Galβ1-4(Fucα1-3)GlcNAcβ-Sp0	40	8
238	Neu5Acα2-3Galβ1-3(6S)GlcNAc-Sp8	40	8
559	Galα1-3Galβ1-4GlcNAcβ1-2Manα1-6(Galα1-3Galβ1-4GlcNAcβ1-2Manα1-3)Manβ1-4GlcNAcβ1-4GlcNAc-Sp24	40	12
417	GαlNacα1-3(Fucα1-2)Galβ1-4(Fucα1-3)GlcNAcβ1-3GalNAc-Sp14	40	7

53	GlcNAcβ1-2Manα1-6(GlcNAcβ1-2Manα1-3)Manβ1-4GlcNAcβ1-4GlcNAcβ-Sp13	40	10
352	Galβ1-4GlcNAcβ1-2Manα1-6(Galβ1-4GlcNAcβ1-2Manα1-3)Manβ1-4GlcNAcβ1-4(Fucα1-6)GlcNAcβ-Sp22	40	8
578	Galβ1-4GlcNAcβ1-3Galβ1-4GlcNAcβ1-3Galβ1-4GlcNAcβ1-3Galβ1-4GlcNAcβ1-2Manα1-6(Galβ1-4GlcNAcβ1-3Galβ1-4GlcNAcβ1-3Galβ1-4GlcNAcβ1-3Galβ1-4GlcNAcβ1-2Manα1-3)Manβ1-4GlcNAcβ1-4(Fucα1-6)GlcNAcβ-Sp24	40	3
111	Galα1-3GαlNacα-Sp16	39	15
484	Galβ1-4GlcNAcβ1-6(Galβ1-4GlcNAcβ1-2)Manα1-6(Galβ1-4GlcNAcβ1-2Manα1-3)Manβ1-4GlcNAcβ1-4(Fucα1-6)GlcNAcβ-Sp24	39	3
9	Neu5Acα-Sp8	39	9
104	Galα1-3(Fucα1-2)Galβ1-4(Fucα1-3)GlcNAcβ-Sp8	38	4
504	Fucα1-2Galβ1-3GlcNAcβ1-6(Fucα1-2Galβ1-3GlcNAcβ1-3)GαlNacα-Sp14	38	12
57	Neu5Acα2-6Galβ1-4GlcNAcβ1-2Manα1-6(Neu5Acα2-6Galβ1-4GlcNAcβ1-2Manα1-3)Manβ1-4GlcNAcβ1-4GlcNAcβ-Sp24	38	6
270	Neu5Acα2-6Galβ1-4GlcNAcβ1-3Galβ1-4(Fucα1-3)GlcNAcβ1-3Galβ1-4(Fucα1-3)GlcNAcβ-Sp0	38	17
253	Neu5Acα2-3Galβ1-4(Fucα1-3)GlcNAcβ1-3Galβ1-4(Fucα1-3)GlcNAcβ1-3Galβ1-4(Fucα1-3)GlcNAcβ-Sp0	37	3
216	Manα1-6(Manα1-3)Manα1-6(Manα1-3)Manβ1-4GlcNAcβ1-4GlcNAcβ-Sp12	37	7
346	Neu5Acα2-6Galβ1-4GlcNAcβ1-2Manα1-6Manβ1-4GlcNAcβ1-4GlcNAc-Sp12	37	23
362	Galα1-3Galβ1-4GlcNAcβ1-2Manα1-6(Galα1-3Galβ1-4GlcNAcβ1-2Manα1-3)Manβ1-4GlcNAcβ1-4GlcNAcβ-Sp20	37	4
370	Galα1-3(Fucα1-2)Galβ1-4GlcNAcβ1-2Manα1-6(Galα1-3(Fucα1-2)Galβ1-4GlcNAcβ1-2Manα1-3)Manβ1-4GlcNAcβ1-4GlcNAcβ-Sp20	37	8
377	Neu5Acα2-3Galβ1-4(Fucα1-3)GlcNAcβ1-3GαlNacα-Sp14	37	5
37	(3S)Galβ1-4GlcNAcβ-Sp8	36	8
341	GlcNAca1-4Galβ1-4GlcNAcβ1-3Galβ1-4(Fucα1-3)GlcNAcβ1-3Galβ1-4(Fucα1-3)GlcNAcβ-Sp0	36	2
378	GalNAcβ1-4GlcNAcβ1-2Manα1-6(GalNAcβ1-4GlcNAcβ1-2Manα1-3)Manβ1-4GlcNAcβ1-4GlcNAc-Sp12	36	1
80	Fucα1-4GlcNAcβ-Sp8	36	9
542	Neu5Gca2-3Galβ1-4GlcNAcβ1-3Galβ1-4GlcNAcβ1-2Manα1-6(Neu5Gca2-3Galβ1-4GlcNAcβ1-3Galβ1-4GlcNAcβ1-2Manα1-3)Manβ1-4GlcNAcβ1-4GlcNAcβ-Sp24	36	2
68	Fucα1-2Galβ1-3GlcNAcβ-Sp8	36	11
425	Fucα1-2Galβ1-3GlcNAcβ1-2Manα1-6(Fucα1-2Galβ1-3GlcNAcβ1-2Manα1-3)Manβ1-4GlcNAcβ1-4(Fucα1-6)GlcNAcβ-Sp22	36	5
25	(3S)Galβ1-4Glcβ-Sp8	35	6
336	Neu5Acα2-3Galβ1-4(Fucα1-3)GlcNAcβ1-6(Neu5Acα2-3Galβ1-3)GalNAc-Sp14	35	5
547	Galβ1-4GlcNAcβ1-3Galβ1-4GlcNAcβ1-3Galβ1-4GlcNAcβ1-2Manα1-6(Galβ1-4GlcNAcβ1-3Galβ1-4GlcNAcβ1-3Galβ1-4GlcNAcβ1-2Manα1-3)Manβ1-4GlcNAcβ1-4GlcNAcβ-Sp24	35	2
97	GalNAcβ1-4(Fucα1-3)GlcNAcβ-Sp0	35	15
103	Galα1-3(Fucα1-2)Galβ1-4(Fucα1-3)GlcNAcβ-Sp0	35	5
124	Galα1-6Glcβ-Sp8	35	5
168	Galβ1-4GlcNAcβ-Sp0	35	4
574	Galβ1-4GlcNAcβ1-3Galβ1-4GlcNAcβ1-2Manα1-6(Galβ1-4GlcNAcβ1-3Galβ1-4GlcNAcβ1-2Manα1-3)Manβ1-4GlcNAcβ1-4(Fucα1-6)GlcNAcβ-Sp24	35	4
528	Neu5Acα2-3Galβ1-3GalNAcβ1-4Galβ1-4Glcβ-Sp0	35	11
63	Fucα1-2Galβ1-3GalNAcβ1-4(Neu5Acα2-3)Galβ1-4Glcβ-Sp0	35	3
79	Fucα1-3GlcNAcβ-Sp8	35	13
172	Galβ1-4Glcβ-Sp8	35	17
3	Manα-Sp8	34	5
42	(6S)Galβ1-4Glcβ-Sp0	34	18
13	Glcβ-Sp8	33	5
60	Fucα1-2Galβ1-3(Fucα1-4)GlcNAcβ-Sp8	33	16
146	Galβ1-3Galβ-Sp8	33	11
402	Galα1-4Galβ1-3GlcNAcβ1-2Manα1-6(Galα1-4Galβ1-3GlcNAcβ1-2Manα1-3)Manβ1-4GlcNAcβ1-4GlcNAcβ-Sp19	33	7
299	Neu5Acα2-3Galβ1-4(Fucα1-3)GlcNAcβ1-6(Galβ1-3)GαlNacα-Sp14	33	4
38	(3S)Galβ-Sp8	32	11
45	(6S)Galβ1-4(6S)Glcβ-Sp8	32	16
70	Fucα1-2Galβ1-4(Fucα1-3)GlcNAcβ1-3Galβ1-4(Fucα1-3)GlcNAcβ1-3Galβ1-4(Fucα1-3)GlcNAcβ-Sp0	32	7
330	Neu5Acα2-3Galβ1-3(Fucα1-4)GlcNAcβ1-3Galβ1-3(Fucα1-4)GlcNAcβ-Sp0	32	5
607	Neu5Acα2-6Galβ1-4GlcNAcβ1-3Galβ1-4GlcNAcβ1-2Manα1-6(Neu5Acα2-6Galβ1-4GlcNAcβ1-3Galβ1-4GlcNAcβ1-2Manα1-3)Manβ1-4GlcNAcβ1-4GlcNAcβ-Sp12	32	2
489	Galβ1-3(Fucα1-4)GlcNAcβ1-6GαlNacα-Sp14	32	7
451	Neu5Acα2-8Neu5Acα2-3Galβ1-3GalNAcβ1-4(Neu5Acα2-8Neu5Acα2-3)Galβ1-4Glcβ-Sp0	32	4
485	Neu5Acα2-3Galβ1-3GlcNAcβ1-2Manα1-6(GlcNAcβ1-4)(Neu5Acα2-3Galβ1-3GlcNAcβ1-2Manα1-3)Manβ1-4GlcNAcβ1-4GlcNAc-Sp21	32	1
32	(3S)Galβ1-4(Fucα1-3)GlcNAc-Sp0	31	7
306	GalNAcβ1-3Galβ-Sp8	31	39

418	Galβ1-4(Fucα1-3)GlcNAcβ1-2Manα1-6(Galβ1-4(Fucα1-3)GlcNAcβ1-2Manα1-3)Manβ1-4GlcNAcβ1-4(Fucα1-6)GlcNAcβ-Sp22	31	4
560	GlcNAcβ1-3Galβ1-4GlcNAcβ1-6(GlcNAcβ1-3Galβ1-3)GαlNacα-Sp14	31	20
114	Galα1-3Galβ1-3GlcNAcβ-Sp0	31	8
93	GαlNacα1-4(Fucα1-2)Galβ1-4GlcNAcβ-Sp8	30	34
181	GlcNAcβ1-3Galβ-Sp8	30	8
78	Fucα1-2Galβ-Sp8	30	7
193	GlcNAcβ1-6GαlNacα-Sp14	30	25
595	Neu5Acα2-3Galβ1-4GlcNAcβ1-3Galβ1-4GlcNAcβ1-6(Neu5Acα2-3Galβ1-4GlcNAcβ1-3Galβ1-4GlcNAcβ1-3)GαlNacα-Sp14	30	8
608	GlcNAcβ1-3Fucα-Sp21	30	26
47	(6S)GlcNAcβ-Sp8	29	8
481	Neu5Acα2-6Galβ1-4GlcNAcβ1-2Manα1-6(Neu5Acα2-6Galβ1-4GlcNAcβ1-2Manα1-3)Manβ1-4GlcNAcβ1-4(Fucα1-6)GlcNAcβ-Sp24	29	4
35	(3S)Galβ1-4(6S)GlcNAcβ-Sp8	29	8
84	(3S)Galβ1-4(Fucα1-3)Glcβ-Sp0	29	8
165	Galβ1-4GlcNAcβ1-3Galβ1-4Glcβ-Sp8	29	6
82	GαlNacα1-3(Fucα1-2)Galβ1-3GlcNAcβ-Sp0	29	14
446	Galβ1-4(Fucα1-3)GlcNAcβ1-6GalNAc-Sp14	29	5
15	GalNAcβ-Sp8	28	5
69	Fucα1-2Galβ1-4(Fucα1-3)GlcNAcβ1-3Galβ1-4(Fucα1-3)GlcNAcβ-Sp0	28	19
150	Galβ1-3GlcNAcβ-Sp8	28	15
499	Fucα1-2Galβ1-3(6S)GlcNAcβ-Sp0	28	6
566	Galβ1-3GlcNAcβ1-6(Galβ1-3)GalNAc-Sp14	28	2
26	(3S)Galβ1-4(6S)Glcβ-Sp0	27	9
59	Fucα1-2Galβ1-3GalNAcβ1-3Galα1-4Galβ1-4Glcβ-Sp9	27	5
61	Fucα1-2Galβ1-3GαlNacα-Sp8	27	4
96	GalNAcβ1-3Galα1-4Galβ1-4GlcNAcβ-Sp0	27	13
118	Galα1-3Galβ-Sp8	27	16
468	Glca1-6Glca1-6Glca1-6Glcβ-Sp10	27	12
65	Fucα1-2Galβ1-3GlcNAcβ1-3Galβ1-4Glcβ-Sp8	27	22
99	GalNAcβ1-4GlcNAcβ-Sp8	27	1
257	Neu5Acα2-3Galβ1-4(Fucα1-3)GlcNAcβ1-3Galβ1-4GlcNAcβ-Sp8	27	6
373	Galα1-3(Fucα1-2)Galβ1-3GlcNAcβ1-2Manα1-6(Galα1-3(Fucα1-2)Galβ1-3GlcNAcβ1-2Manα1-3)Manβ1-4GlcNAcβ1-4GlcNAcβ-Sp20	27	6
226	GalNAcβ1-4(Neu5Acα2-8Neu5Acα2-8Neu5Acα2-3)Galβ1-4Glcβ-Sp0	26	6
263	Neu5Acα2-3Galβ1-4Glcβ-Sp0	26	4
142	Galβ1-3GalNAcβ-Sp8	26	18
145	Galβ1-3GalNAcβ1-4Galβ1-4Glcβ-Sp8	26	4
335	GαlNacα1-3(Fucα1-2)Galβ1-4GlcNAcβ1-3Galβ1-4GlcNAcβ1-3Galβ1-4GlcNAcβ-Sp0	26	3
365	Neu5Acα2-6GlcNAcβ1-4GlcNAc-Sp21	26	53
312	MurNAcβ1-4GlcNAcβ-Sp10	26	16
48	Neu5,9Ac$_2$a-Sp8	26	5
113	Galα1-3Galβ1-4(Fucα1-3)GlcNAcβ-Sp8	26	10
372	GαlNacα1-3(Fucα1-2)Galβ1-3GlcNAcβ1-2Manα1-6(GαlNacα1-3(Fucα1-2)Galβ1-3GlcNAcβ1-2Manα1-3)Manβ1-4GlcNAcβ1-4GlcNAcβ-Sp20	26	5
457	Galβ1-4GlcNAcβ1-6(Galβ1-4GlcNAcβ1-2)Manα1-6(Galβ1-4GlcNAcβ1-2Manα1-3)Manβ1-4GlcNAcβ1-4GlcNAcβ-Sp19	26	7
449	Galα1-3(Fucα1-2)Galβ1-4GlcNAcβ1-6(Galα1-3(Fucα1-2)Galβ1-4GlcNAcβ1-3)GalNAc-Sp14	25	9
277	Galβ1-3(Fucα1-4)GlcNAcβ1-3Galβ1-3(Fucα1-4)GlcNAcβ-Sp0	25	4
423	GαlNacα1-3(Fucα1-2)Galβ1-3GlcNAcβ1-3GalNAc-Sp14	25	5
524	Galα1-3(Fucα1-2)Galβ1-3GlcNAcβ1-6GalNAc-Sp14	25	17
169	Galβ1-4GlcNAcβ-Sp8	25	8
248	Fucα1-2(6S)Galβ1-4Glcβ-Sp0	25	7
273	Neu5Acα2-6Galβ1-4Glcβ-Sp8	25	4
29	(3S)Galβ1-3GαlNacα-Sp8	25	8
31	(3S)Galβ1-3GlcNAcβ-Sp8	25	7
121	Galα1-4Galβ1-4GlcNAcβ-Sp8	25	19
436	Galβ1-4GlcNAcβ1-6(Galβ1-4GlcNAcβ1-2)Manα1-6(GlcNAcβ1-4)(Galβ1-4GlcNAcβ1-2Manα1-3)Manβ1-4GlcNAcβ1-4GlcNAc-Sp21	25	10

507	Galβ1-4GlcNAcβ1-6(Galβ1-4GlcNAcβ1-2)Manα1-6(GlcNAcβ1-4)Galβ1-4GlcNAcβ1-4(Gal β1-4GlcNAcβ1-2)Manα1-3)Manβ1-4GlcNAcβ1-4(Fucα1-6)GlcNAc-Sp21	25	8
117	Galα1-3Galβ1-4Glc-Sp10	24	4
122	Galα1-4Galβ1-4Glcβ-Sp0	24	6
434	Galβ1-4GlcNAcβ1-2Manα1-6(GlcNAcβ1-4)(Galβ1-4GlcNAcβ1-2Manα1-3)Manβ1-4GlcNAcβ1-4GlcNAc-Sp21	24	13
460	Neu5Acα2-3Galβ1-4GlcNAcβ1-6(Neu5Acα2-3Galβ1-4GlcNAcβ1-2)Manα1-6(GlcNAcβ1-4)(Neu5Acα2-3Galβ1-4GlcNAcβ1-2Manα1-3)Manβ1-4GlcNAcβ1-4GlcNAcβ-Sp21	24	8
36	(3S)Galβ1-4GlcNAcβ-Sp0	24	6
49	Neu5,9Ac2a2-6Galβ1-4GlcNAcβ-Sp8	24	4
355	KDNa2-3Galβ1-4(Fucα1-3)GlcNAc-Sp0	24	6
27	(3S)Galβ1-4(6S)Glcβ-Sp8	24	2
141	Galβ1-3GαlNacα-Sp16	24	6
443	(6S)Galβ1-3(6S)GlcNAc-Sp0	24	2
127	Galβ1-3GlcNAcβ1-3Galβ1-4(Fucα1-3)GlcNAcβ-Sp0	24	23
204	GlcAβ1-6Galβ-Sp8	24	2
358	KDNa2-3Galβ1-3GαlNacα-Sp14	24	12
452	GalNAcβ1-4Galβ1-4Glcβ-Sp0	24	2
482	Neu5Acα2-3Galβ1-4GlcNAcβ1-2Manα1-6(Neu5Acα2-3Galβ1-4GlcNAcβ1-2Manα1-3)Manβ1-4GlcNAcβ1-4(Fucα1-6)GlcNAcβ-Sp24	23	9
120	Galα1-4Galβ1-4GlcNAcβ-Sp0	23	10
430	GlcNAcβ1-2Manα1-6(GlcNAcβ1-4)(GlcNAcβ1-2Manα1-3)Manβ1-4GlcNAcβ1-4GlcNAc-Sp21	23	14
495	Galβ1-4(Fucα1-3)GlcNAcβ1-2Manα-Sp0	23	5
344	Neu5Acα2-6Galβ1-4GlcNAcβ1-2Manα1-6(Manα1-3)Manβ1-4GlcNAcβ1-4GlcNAc-Sp12	23	2
491	(3S)Galβ1-3(Fucα1-4)GlcNAcβ-Sp0	23	5
493	Fucα1-2Galβ1-4GlcNAcβ1-6GαlNacα-Sp14	23	4
40	(4S)Galβ1-4GlcNAcβ-Sp8	23	3
106	Galα1-3(Fucα1-2)Galβ1-4Glcβ-Sp0	23	8
498	Fucα1-2Galβ1-4GlcNAcβ1-2Manα-Sp0	23	6
112	Galα1-3GalNAcβ-Sp8	22	4
383	Galβ1-4GlcNAcβ1-6(Fucα1-4(Fucα1-2Galβ1-3)GlcNAcβ1-3)Galβ1-4Glc-Sp21	22	14
479	Neu5Acα2-6Galβ1-4GlcNAcβ1-6GαlNacα-Sp14	22	2
52	GlcNAcβ1-2Manα1-6(GlcNAcβ1-2Manα1-3)Manβ1-4GlcNAcβ1-4GlcNAcβ-Sp12	22	4
242	Neu5Acα2-3Galβ1-3(6S)GαlNacα-Sp8	22	2
72	Fucα1-2Galβ1-4(Fucα1-3)GlcNAcβ-Sp8	22	3
284	Neu5Gca2-3Galβ1-4Glcβ-Sp0	22	4
515	(4S)GalNAcβ-Sp10	22	2
600	Neu5Acα2-6Galβ1-4GlcNAcβ1-3Galβ1-4GlcNAcβ1-6(Galβ1-3)GαlNacα-Sp14	22	6
75	Fucα1-2Galβ1-4GlcNAcβ-Sp0	22	3
130	Fucα1-4(Galβ1-3)GlcNAcβ-Sp8	22	2
161	Galβ1-4GlcNAcβ1-3Galβ1-4(Fucα1-3)GlcNAcβ1-3Galβ1-4(Fucα1-3)GlcNAcβ-Sp0	22	3
188	GlcNAcβ1-4Galβ1-4GlcNAcβ-Sp8	22	3
435	Galβ1-4GlcNAcβ1-2Manα1-6(GlcNAcβ1-4)(Galβ1-4GlcNAcβ1-4(Galβ1-4GlcNAcβ1-2)Manα1-3)Manβ1-4GlcNAcβ1-4GlcNAc-Sp21	22	4
4	GαlNacα-Sp8	21	5
219	(3S)Galβ1-4(Fucα1-3)(6S)GlcNAcβ-Sp8	21	1
521	Galα1-3(Fucα1-2)Galβ1-4GlcNAcβ1-2Manα-Sp0	21	3
20	Galβ1-4GlcNAcβ1-6(Galβ1-4GlcNAcβ1-3)GalNAc-Sp14	21	6
197	Glca1-6Glca1-6Glcβ-Sp8	21	3
420	GlcNAcβ1-2(GlcNAcβ1-6)Manα1-6(GlcNAcβ1-2Manα1-3)Manβ1-4GlcNAcβ1-4GlcNAcβ-Sp19	21	3
520	Galα1-3Galβ1-4GlcNAcβ1-2Manα-Sp0	21	3
34	(3S)Galβ1-4(6S)GlcNAcβ-Sp0	21	7
67	Fucα1-2Galβ1-3GlcNAcβ-Sp0	21	16
101	Galα1-3(Fucα1-2)Galβ1-3GlcNAcβ-Sp0	21	3
246	Neu5Acα2-3Galβ1-3GalNAcβ1-3Galα1-4Galβ1-4Glcβ-Sp0	21	2
601	Neu5Acα2-6Galβ1-4GlcNAcβ1-6(Galβ1-3)GαlNacα-Sp14	21	20
41	(6P)Manα-Sp8	21	10
128	Galβ1-3(Fucα1-4)GlcNAc-Sp0	21	4

129	Galβ1-3(Fucα1-4)GlcNAc-Sp8	21	5
416	Galα1-3(Fucα1-2)Galβ1-4(Fucα1-3)GlcNAcβ1-3GalNAc-Sp14	21	4
123	Galα1-4GlcNAcβ-Sp8	20	4
140	Galβ1-3GαlNacα-Sp14	20	4
408	Galα1-3(Fucα1-2)Galβ1-4(Fucα1-3)Glcβ-Sp21	20	2
599	Neu5Acα2-3Galβ1-4GlcNAcβ1-3Galβ1-4GlcNAcβ1-6(Galβ1-3)GαlNacα-Sp14	20	5
22	6S(3S)Galβ1-4(6S)GlcNAcβ-Sp0	20	2
88	GlcNAcβ1-3Galβ1-3GαlNacα-Sp8	20	2
125	Galβ1-2Galβ-Sp8	20	14
196	Glca1-4Glca-Sp8	20	1
100	Galα1-2Galβ-Sp8	20	8
133	GlcNAcβ1-6(Galβ1-3)GαlNacα-Sp8	20	11
313	Manα1-6Manβ-Sp10	20	2
343	GlcNAca1-4Galβ1-3GalNAc-Sp14	20	13
502	GalNAcβ1-4(Fucα1-3)(6S)GlcNAcβ-Sp8	20	3
11	Neu5Acβ-Sp8	20	16
71	Fucα1-2Galβ1-4(Fucα1-3)GlcNAcβ-Sp0	20	6
98	GalNAcβ1-4GlcNAcβ-Sp0	20	5
119	Galα1-4(Fucα1-2)Galβ1-4GlcNAcβ-Sp8	20	14
156	Galβ1-4(6S)Glcβ-Sp8	20	10
286	Neu5Gca2-6Galβ1-4GlcNAcβ-Sp0	20	6
298	(6P)Glcβ-Sp10	20	8
503	(3S)GalNAcβ1-4(Fucα1-3)GlcNAcβ-Sp8	20	4
90	GαlNacα1-3(Fucα1-2)Galβ-Sp18	19	3
23	6S(3S)Galβ1-4GlcNAcβ-Sp0	19	7
89	GαlNacα1-3(Fucα1-2)Galβ-Sp8	19	5
91	GαlNacα1-3GalNAcβ-Sp8	19	2
171	Galβ1-4Glcβ-Sp0	19	3
448	Fucα1-2Galβ1-4GlcNAcβ1-6(Fucα1-2Galβ1-4GlcNAcβ1-3)GalNAc-Sp14	19	12
16	GlcNAcβ-Sp0	19	5
152	Galβ1-4(Fucα1-3)GlcNAcβ-Sp8	19	4
170	Galβ1-4GlcNAcβ-Sp23	19	2
297	(6S)Galβ1-4(6S)GlcNAcβ-Sp0	19	3
375	Neu5Acα2-3Galβ1-4GlcNAcβ1-3GalNAc-Sp14	19	2
439	Galβ1-6Galβ-Sp10	19	5
66	Fucα1-2Galβ1-3GlcNAcβ1-3Galβ1-4Glcβ-Sp10	18	8
348	Galβ1-4GlcNAcβ1-2Manα1-3Manβ1-4GlcNAcβ1-4GlcNAc-Sp12	18	7
167	Galβ1-4GlcNAcβ1-6(Galβ1-3)GalNAc-Sp14	18	2
176	GlcNAcβ1-6(GlcNAcβ1-3)GαlNacα-Sp8	18	4
105	Galα1-3(Fucα1-2)Galβ1-4GlcNAc-Sp0	18	1
108	Galα1-3(Fucα1-2)Galβ-Sp18	18	3
132	Galβ1-4GlcNAcβ1-6GalNAc-Sp14	18	9
536	Galα1-3(Fucα1-2)Galβ1-3GalNAcβ1-3Galα1-4Galβ1-4Glc-Sp21	18	4
192	GlcNAcβ1-6GαlNacα-Sp8	18	3
429	Fucα1-3GlcNAcβ1-6(Galβ1-4GlcNAcβ1-3)Galβ1-4Glc-Sp21	18	5
92	GαlNacα1-3Galβ-Sp8	17	4
157	Galβ1-4GαlNacα1-3(Fucα1-2)Galβ1-4GlcNAcβ-Sp8	17	6
220	Fucα1-2(6S)Galβ1-4GlcNAcβ-Sp0	17	5
322	Neu5Gcβ2-6Galβ1-4GlcNAc-Sp8	17	6
388	Fucα1-2Galβ1-3GαlNacα1-3(Fucα1-2)Galβ1-4Glcβ-Sp0	17	2
496	Fucα1-2(6S)Galβ1-3GlcNAcβ-Sp0	17	3
30	(3S)Galβ1-3GlcNAcβ-Sp0	17	9
58	Fucα1-2Galβ1-3GalNAcβ1-3Galα-Sp9	17	1

126	Galβ1-3(Fucα1-4)GlcNAcβ1-3Galβ1-4(Fucα1-3)GlcNAcβ-Sp0	17	8
349	Galβ1-4GlcNAcβ1-2Manα1-6Manβ1-4GlcNAcβ1-4GlcNAc-Sp12	17	6
467	Galα1-3(Fucα1-2)Galβ1-3GalNAcβ-Sp8	17	5
537	Galβ1-3GalNAcβ1-3Gal-Sp21	17	6
281	Neu5Gca2-3Galβ1-3GlcNAcβ-Sp0	17	12
287	Neu5Gca-Sp8	17	2
332	Galα1-4Galβ1-4GlcNAcβ1-3Galβ1-4Glcβ-Sp0	17	11
469	Glca1-4Glca1-4Glca1-4Glcβ-Sp10	17	4
497	Galα1-3(Fucα1-2)Galβ1-4GlcNAcβ1-6GαlNacα-Sp14	17	3
8	Rhaa-Sp8	16	12
77	Fucα1-2Galβ1-4Glcβ-Sp0	16	7
86	GαlNacα1-3(Fucα1-2)Galβ1-4GlcNAcβ-Sp8	16	2
154	Galβ1-4(Fucα1-3)GlcNAcβ1-3Galβ1-4(Fucα1-3)GlcNAcβ1-3Galβ1-4(Fucα1-3)GlcNAcβ-Sp0	16	5
239	Neu5Acα2-3Galβ1-3(Fucα1-4)GlcNAcβ-Sp8	16	4
342	GlcNAca1-4Galβ1-4GlcNAcβ1-3Galβ1-4GlcNAcβ-Sp0	16	11
411	Neu5Acα2-3Galβ1-3GalNAcβ1-4(Neu5Acα2-8Neu5Acα2-3)Galβ1-4Glcβ-Sp0	16	5
592	GlcNAcβ1-3Galβ1-4GlcNAcβ1-3GαlNacα-Sp14	16	15
185	GlcNAcβ1-3Galβ1-4Glcβ-Sp0	16	4
357	KDNa2-3Galβ1-4Glc-Sp0	16	9
189	GlcNAcβ1-4GlcNAcβ1-4GlcNAcβ1-4GlcNAcβ1-4GlcNAcβ1-4GlcNAcβ1-Sp8	16	7
203	GlcAβ1-3Galβ-Sp8	16	5
354	(6S)GlcNAcβ1-3Galβ1-4GlcNAcβ-Sp0	16	7
110	Galα1-3GαlNacα-Sp8	16	9
116	Galα1-3Galβ1-4Glcβ-Sp0	16	7
151	Galβ1-4(Fucα1-3)GlcNAcβ-Sp0	16	6
249	Neu5Acα2-3Galβ1-3GlcNAcβ-Sp0	16	2
565	GlcNAβ1-3Galβ1-3GalNAc-Sp14	16	8
73	Fucα1-2Galβ1-4GlcNAcβ1-3Galβ1-4GlcNAcβ-Sp0	15	5
74	Fucα1-2Galβ1-4GlcNAcβ1-3Galβ1-4GlcNAcβ1-3Galβ1-4GlcNAcβ-Sp0	15	4
76	Fucα1-2Galβ1-4GlcNAcβ-Sp8	15	5
107	Galα1-3(Fucα1-2)Galβ-Sp8	15	3
237	Neu5Acα2-3GalNAcβ1-4GlcNAcβ-Sp0	15	13
526	Galα1-3Galβ1-3GlcNAcβ1-2Manα-Sp0	15	5
85	GαlNacα1-3(Fucα1-2)Galβ1-4GlcNAcβ-Sp0	15	7
139	Galβ1-3GαlNacα-Sp8	15	16
462	Neu5Acα2-6Galβ1-4GlcNAcβ1-2Manα1-6(GlcNAcβ1-4)(Neu5Acα2-6Galβ1-4GlcNAcβ1-2Manα1-3)Manβ1-4GlcNAcβ1-4GlcNAcβ-Sp21	15	2
55	Neu5Acα2-6Galβ1-4GlcNAcβ1-2Manα1-6(Neu5Acα2-6Galβ1-4GlcNAcβ1-2Manα1-3)Manβ1-4GlcNAcβ1-4GlcNAcβ-Sp12	15	9
94	GalNAcβ1-3GαlNacα-Sp8	15	9
232	GalNAcβ1-4(Neu5Acα2-3)Galβ1-4GlcNAcβ-Sp8	15	7
296	4S(3S)Galβ1-4GlcNAcβ-Sp0	15	10
329	Neu5Acα2-6Galβ1-4GlcNAcβ1-3Galβ1-3GlcNAcβ-Sp0	15	10
609	Galβ1-3GalNAcβ1-4(Neu5Acα2-8Neu5Acα2-8Neu5Acα2-3)Galβ1-4Glcβ-Sp21	15	8
400	Galβ1-4(Fucα1-3)GlcNAcβ1-3GαlNacα-Sp14	15	5
437	Galβ1-4GlcNAcβ1-6(Galβ1-4GlcNAcβ1-2)Manα1-6(GlcNAcβ1-4)(Galβ1-4GlcNAcβ1-4(Galβ1-4GlcNAcβ1-2)Manα1-3)Manβ1-4GlcNAcβ1-4GlcNAc-Sp21	15	6
527	GalNAcβ1-4GlcNAcβ1-2Manα-Sp0	15	3
279	Neu5Acβ2-6Galβ1-4GlcNAcβ-Sp8	14	4
310	GlcNAcβ1-4GlcNAcβ-Sp10	14	4
321	Neu5Acα2-8Neu5Acα2-8Neu5Acβ-Sp8	14	6
422	Galα1-3(Fucα1-2)Galβ1-3GlcNAcβ1-3GalNAc-Sp14	14	3
464	Neu5Acα2-6Galβ1-4GlcNAcβ1-6(Neu5Acα2-6Galβ1-4GlcNAcβ1-2)Manα1-6(GlcNAcβ1-4)(Neu5Acα2-6Galβ1-4GlcNAcβ1-2Manα1-3)Manβ1-4GlcNAcβ1-4GlcNAcβ-Sp21	14	4
33	(3S)Galβ1-4(Fucα1-3)GlcNAc-Sp8	14	10
62	Fucα1-2Galβ1-3GαlNacα-Sp14	14	8
179	GlcNAcβ1-3GαlNacα-Sp8	14	7

180	GlcNAcβ1-3GαlNacα-Sp14	14	9
206	KDNa2-3Galβ1-4GlcNAcβ-Sp0	14	18
509	Galβ1-3(6S)GlcNAcβ-Sp8	14	1
43	(6S)Galβ1-4Glcβ-Sp8	14	11
46	Neu5Acα2-3(6S)Galβ1-4GlcNAcβ-Sp8	14	6
338	GlcNAca1-4Galβ1-4GlcNAcβ-Sp0	14	7
512	(3S)GalNAcβ1-4(3S)GlcNAc-Sp8	14	6
522	GαlNacα1-3(Fucα1-2)Galβ1-4 GlcNAcβ1-2Manα-Sp0	14	13
535	GαlNacα1-3(Fucα1-2)Galβ1-3GalNAcβ1-3Galα1-4Galβ1-4Glc-Sp21	14	3
486	Neu5Acα2-6Galβ1-4GlcNAcβ1-6(Fucα1-2Galβ1-4(Fucα1-3)GlcNAcβ1-3)Galβ1-4Glc-Sp21	14	1
555	Neu5Gca2-8Neu5Gca2-6Galβ1-4GlcNAc-Sp0	13	6
268	Neu5Acα2-6Galβ1-4GlcNAcβ-Sp0	13	10
271	Neu5Acα2-6Galβ1-4GlcNAcβ1-3Galβ1-4GlcNAcβ-Sp0	13	12
525	Neu5Acα2-3Galβ1-3GlcNAcβ1-2Manα-Sp0	13	10
148	Galβ1-3GlcNAcβ1-3Galβ1-4Glcβ-Sp10	13	5
182	GlcNAcβ1-3Galβ1-4GlcNAcβ-Sp0	13	2
265	Neu5Acα2-6GαlNacα-Sp8	13	7
407	GalNAcβ1-3Galα1-6Galβ1-4Glcβ-Sp8	13	7
490	Neu5Acα2-3Galβ1-3GlcNAcβ1-6GαlNacα-Sp14	13	6
514	(3S)GalNAcβ1-4GlcNAc-Sp8	13	8
218	Neu5Acα2-3Galβ1-4GlcNAcβ1-3Galβ1-4(Fucα1-3)GlcNAcβ-Sp0	13	4
228	GalNAcβ1-4(Neu5Acα2-8Neu5Acα2-3)Galβ1-4Glcβ-Sp0	13	6
245	Neu5Acα2-3Galβ-Sp8	13	9
356	KDNa2-6Galβ1-4GlcNAc-Sp0	13	2
405	Galβ1-3GlcNAcβ1-6Galβ1-4GlcNAcβ-Sp0	13	10
95	GalNAcβ1-3(Fucα1-2)Galβ-Sp8	12	7
234	Neu5Acα2-3Galβ1-3GalNAcβ1-4(Neu5Acα2-3)Galβ1-4Glcβ-Sp0	12	11
381	Galβ1-3GlcNAcβ1-3Galβ1-4GlcNAcβ1-6(Galβ1-3GlcNAcβ1-3)Galβ1-4Glcβ-Sp0	12	10
399	Fucα1-2Galβ1-4GlcNAcβ1-3GαlNacα-Sp14	12	6
409	Galβ1-4GlcNAcβ1-6(Neu5Acα2-6Galβ1-3GlcNAcβ1-3)Galβ1-4Glc-Sp21	12	4
413	GαlNacα1-3(Fucα1-2)Galβ1-4GlcNAcβ1-3GαlNacα-Sp14	12	4
164	Galβ1-4GlcNAcβ1-3Galβ1-4Glcβ-Sp0	12	10
492	Galβ1-4(Fucα1-3)GlcNAcβ1-6(Neu5Acα2-6(Neu5Acα2-3Galβ1-3)GlcNAcβ1-3)Galβ1-4Glc-Sp21	12	4
264	Neu5Acα2-3Galβ1-4Glcβ-Sp8	12	4
267	Neu5Acα2-6Galβ1-4(6S)GlcNAcβ-Sp8	12	21
320	Neu5Acα2-8Neu5Acβ-Sp17	12	5
233	GalNAcβ1-4(Neu5Acα2-3)Galβ1-4Glcβ-Sp0	12	9
260	Neu5Acα2-3Galβ1-4GlcNAcβ-Sp8	12	9
561	GalNAcβ1-3GlcNAcβ-Sp0	12	2
177	GlcNAcβ1-6(GlcNAcβ1-3)GαlNacα-Sp14	11	17
289	Galβ1-3GlcNAcβ1-3Galβ1-3GlcNAcβ-Sp0	11	8
384	Galβ1-4(Fucα1-3)GlcNAcβ1-6(Fucα1-4(Fucα1-2Galβ1-3)GlcNAcβ1-3)Galβ1-4Glc-Sp21	11	7
466	Galα1-3(Fucα1-2)Galβ1-3GαlNacα-Sp8	11	7
519	Neu5Acα2-6Galβ1-4GlcNAcβ1-2Man-Sp0	11	9
225	GalNAcβ1-4(Neu5Acα2-8Neu5Acα2-8Neu5Acα2-8Neu5Acα2-3)Galβ1-4Glcβ-Sp0	11	4
331	Neu5Acα2-6Galβ1-4GlcNAcβ1-3Galβ1-4GlcNAcβ1-3Galβ1-4GlcNAcβ-Sp0	11	3
455	Neu5Acα2-6Galβ1-4GlcNAcβ1-6(Fucα1-2Galβ1-3GlcNAcβ1-3)Galβ1-4Glc-Sp21	11	4
510	(6S)(4S)GalNAcβ1-4GlcNAc-Sp8	11	7
591	Neu5Acα2-3Galβ1-4GlcNAcβ1-3Galβ1-4GlcNAcβ1-3GαlNacα-Sp14	11	8
594	GlcNAcβ1-3Galβ1-4GlcNAcβ1-6(GlcNAcβ1-3Galβ1-4GlcNAcβ1-3)GαlNacα-Sp14	11	5
325	Neu5Acα2-3Galβ1-4GlcNAcβ1-2Manα1-6(Neu5Acα2-6Galβ1-4GlcNAcβ1-2Manα1-3)Manβ1-4GlcNAcβ1-4GlcNAcβ-Sp12	11	5
143	Galβ1-3GalNAcβ1-3Galα1-4Galβ1-4Glcβ-Sp0	11	9
231	GalNAcβ1-4(Neu5Acα2-3)Galβ1-4GlcNAcβ-Sp0	11	7

250	Neu5Acα2-3Galβ1-3GlcNAcβ-Sp8	11	9
302	Galβ1-4GlcNAcβ1-6(Galβ1-4GlcNAcβ1-3)Galβ1-4GlcNAc-Sp0	11	2
398	Neu5Acα2-3Galβ1-3GlcNAcβ1-3GαlNacα-Sp14	10	3
421	Fucα1-2Galβ1-3GlcNAcβ1-3GalNAc-Sp14	10	6
476	Neu5Acα2-6Galβ1-4GlcNAcβ1-6(Galβ1-3GlcNAcβ1-3)Galβ1-4Glcβ-Sp21	10	1
506	GlcNAcβ1-6(GlcNAcβ1-2)Manα1-6(GlcNAcβ1-4)(GlcNAcβ1-4(GlcNAcβ1-2)Manα1-3)Manβ1-4GlcNAcβ1-4(Fucα1-6)GlcNAc-Sp21	10	5
516	Galβ1-4(6P)GlcNAcβ-Sp0	10	5
5	GαlNacα-Sp15	10	7
324	Neu5Acα2-3Galβ1-4GlcNAcβ1-2Manα1-6(Neu5Acα2-3Galβ1-4GlcNAcβ1-2Manα1-3)Manβ1-4GlcNAcβ1-4GlcNAcβ-Sp12	10	3
39	(6S)(4S)Galβ1-4GlcNAcβ-Sp0	10	6
159	Galβ1-4GlcNAcβ1-3GαlNacα-Sp8	10	4
173	GlcNAca1-3Galβ1-4GlcNAcβ-Sp8	10	6
190	GlcNAcβ1-4GlcNAcβ1-4GlcNAcβ1-4GlcNAcβ1-4GlcNAcβ1-Sp8	10	10
382	Galβ1-4(Fucα1-3)GlcNAcβ1-6(Galβ1-3GlcNAcβ1-3)Galβ1-4Glc-Sp21	10	5
160	Galβ1-4GlcNAcβ1-3GalNAc-Sp14	10	6
259	Neu5Acα2-3Galβ1-4GlcNAcβ-Sp0	10	6
275	Neu5Acα2-8Neu5Acα-Sp8	10	4
307	GlcAβ1-3GlcNAcβ-Sp8	10	4
501	Neu5Acα2-6GalNAcβ1-4(6S)GlcNAcβ-Sp8	10	2
508	Galβ1-3GlcNAca1-3Galβ1-4GlcNAcβ-Sp8	10	3
569	(3S)GlcAβ1-3Galβ1-4GlcNAcβ1-2Manα-Sp0	10	3
572	Neu5Acα2-8Neu5Acα2-3Galβ1-3GalNAcβ1-4(Neu5Acα2-3)Galβ1-4Glc-Sp21	10	4
10	Neu5Acα-Sp11	9	5
56	Neu5Acα2-6Galβ1-4GlcNAcβ1-2Manα1-6(Neu5Acα2-6Galβ1-4GlcNAcβ1-2Man-a1-3)Manβ1-4GlcNAcβ1-4GlcNAcβ-Sp21	9	6
87	GαlNacα1-3(Fucα1-2)Galβ1-4Glcβ-Sp0	9	10
137	Neu5Acβ2-6(Galβ1-3)GαlNacα-Sp8	9	5
386	Galβ1-4GlcNAcβ1-6(Galβ1-4GlcNAcβ1-2)Manα1-6(Galβ1-4GlcNAcβ1-4(Galβ1-4GlcNAcβ1-2)Manα1-3)Manβ1-4GlcNAcβ1-4GlcNAcβ-Sp21	9	4
387	GlcNAcβ1-2Manα1-6(GlcNAcβ1-4(GlcNAcβ1-2)Manα1-3)Manβ1-4GlcNAcβ1-4GlcNAc-Sp21	9	2
438	Galβ1-4Galβ-Sp10	9	7
487	Galβ1-3GlcNAcβ1-6GαlNacα-Sp14	9	7
19	Galβ1-4GlcNAcβ1-6(Galβ1-4GlcNAcβ1-3)GαlNacα-Sp8	9	9
115	Galα1-3Galβ1-4GlcNAcβ-Sp8	9	6
301	Neu5Acα2-6Galβ1-4GlcNAcβ1-2Manα1-6(Galβ1-4GlcNAcβ1-2Manα1-3)Manβ1-4GlcNAcβ1-4GlcNAcβ-Sp12	9	2
488	Galα1-3Galβ1-3GlcNAcβ1-6GαlNacα-Sp14	9	2
494	Galα1-3Galβ1-4GlcNAcβ1-6GαlNacα-Sp14	9	1
530	Galβ1-4GlcNAcβ1-2 Manα1-6(GlcNAcβ1-4)(Galβ1-4GlcNAcβ1-2Manα1-3)Manβ1-4GlcNAcβ1-4(Fucα1-6)GlcNAc-Sp21	9	2
175	GlcNAcβ1-2Galβ1-3GαlNacα-Sp8	9	3
261	Neu5Acα2-3Galβ1-4GlcNAcβ1-3Galβ1-4GlcNAcβ-Sp0	9	3
389	Fucα1-2Galβ1-3GαlNacα1-3(Fucα1-2)Galβ1-4GlcNAcβ-Sp0	9	2
441	GalNAcβ1-6GalNAcβ-Sp8	9	4
269	Neu5Acα2-6Galβ1-4GlcNAcβ-Sp8	9	6
339	GlcNAca1-4Galβ1-3GlcNAcβ-Sp0	9	6
162	Galβ1-4GlcNAcβ1-3Galβ1-4GlcNAcβ1-3Galβ1-4GlcNAcβ-Sp0	8	2
258	Neu5Acα2-3Galβ1-4GlcNAcβ1-3Galβ1-4GlcNAcβ1-3Galβ1-4GlcNAcβ-Sp0	8	11
385	Galβ1-3GlcNAcβ1-3Galβ1-4(Fucα1-3)GlcNAcβ1-6(Galβ1-3GlcNAcβ1-3)Galβ1-4Glc-Sp21	8	7
397	Galβ1-4GlcNAcβ1-2Manα1-6(GlcNAcβ1-2Manα1-3)Manβ1-4GlcNAcβ1-4GlcNAc-Sp12	8	2
424	Galα1-3Galβ1-3GlcNAcβ1-3GalNAc-Sp14	8	2
551	Neu5Gca2-8Neu5Gca2-3Galβ1-4GlcNAc-Sp0	8	4
596	Neu5Acα2-6Galβ1-4GlcNAcβ1-3Galβ1-4GlcNAcβ1-3GαlNacα-Sp14	8	3
44	(6S)Galβ1-4GlcNAcβ-Sp8	8	6
252	Neu5Acα2-3Galβ1-4(Fucα1-3)(6S)GlcNAcβ-Sp8	8	4
266	Neu5Acα2-6GalNAcβ1-4GlcNAcβ-Sp0	8	6
272	Neu5Acα2-6Galβ1-4Glcβ-Sp0	8	5

465	Neu5Acα2-6Galβ1-4GlcNAcβ1-6(Neu5Acα2-6Galβ1-4GlcNAcβ1-2)Manα1-6(GlcNAcβ1-4)(Neu5Acα2-6Galβ1-4GlcNAcβ1-4(Neu5Acα2-6Galβ1-4GlcNAcβ1-2)Manα1-3)Manβ1-4GlcNAcβ1-4GlcNAcβ-Sp21	8	1
470	Neu5Acα2-3Galβ1-4GlcNAcβ1-6(Neu5Acα2-3Galβ1-4GlcNAcβ1-3)GαlNacα-Sp14	8	3
480	Neu5Acα2-6Galβ1-4 GlcNAcβ1-6(Neu5Acα2-6Galβ1-4GlcNAcβ1-3)GαlNacα-Sp14	8	4
531	Galβ1-4GlcNAcβ1-2 Manα1-6(Galβ1-4GlcNAcβ1-4)(Galβ1-4GlcNAcβ1-2Manα1-3)Manβ1-4GlcNAcβ1-4(Fucα1-6)GlcNAc-Sp21	8	2
230	Neu5Acα2-3(6S)Galβ1-4(Fucα1-3)GlcNAcβ-Sp8	8	7
254	Neu5Acα2-3Galβ1-4(Fucα1-3)GlcNAcβ-Sp0	8	8
283	Neu5Gca2-3Galβ1-4GlcNAcβ-Sp0	8	2
311	GlcNAcβ1-4GlcNAcβ-Sp12	8	5
333	GalNAcβ1-3Galα1-4Galβ1-4GlcNAcβ1-3Galβ1-4Glcβ-Sp0	8	4
458	Neu5Acα2-3Galβ1-4GlcNAcβ1-2Manα1-6(GlcNAcβ1-4)(Neu5Acα2-3Galβ1-4GlcNAcβ1-2Manα1-3)Manβ1-4GlcNAcβ1-4GlcNAcβ-Sp21	8	4
463	Neu5Acα2-6Galβ1-4GlcNAcβ1-4Manα1-6(GlcNAcβ1-4)(Neu5Acα2-6Galβ1-4GlcNAcβ1-4(Neu5Acα2-6Galβ1-4GlcNAcβ1-2)Manα1-3)Manβ1-4GlcNAcβ1-4GlcNAcβ-Sp21	8	1
533	Neu5Acα2-3Galβ1-4(Fucα1-3)GlcNAcβ1-2Manα-Sp0	8	5
603	GlcNAcβ1-6(Neu5Acα2-3Galβ1-3)GαlNacα-Sp14	8	6
138	Neu5Acα2-6(Galβ1-3)GlcNAcβ1-4Galβ1-4Glcβ-Sp10	7	4
274	Neu5Acα2-6Galβ-Sp8	7	3
294	Neu5Acα2-3Galβ1-3GlcNAcβ1-3Galβ1-3GlcNAcβ-Sp0	7	4
553	Neu5Gca2-8Neu5Acα2-3Galβ1-4GlcNAc-Sp0	7	5
147	Galβ1-3GlcNAcβ1-3Galβ1-4GlcNAcβ-Sp0	7	8
229	Neu5Acα2-8Neu5Acα2-8Neu5Acα-Sp8	7	8
379	Galβ1-3GαlNacα1-3(Fucα1-2)Galβ1-4Glc-Sp0	7	3
442	(6S)Galβ1-3GlcNAcβ-Sp0	7	3
534	GlcNAcβ1-3Galβ1-4GlcNAcβ1-6(GlcNAcβ1-3)Galβ1-4GlcNAc-Sp0	7	1
131	Galβ1-4GlcNAcβ1-6GαlNacα-Sp8	7	11
247	Neu5Acα2-3Galβ1-3GlcNAcβ1-3Galβ1-4GlcNAcβ-Sp0	7	1
317	Neu5Acα2-3Galβ1-4GlcNAcβ1-6(Neu5Acα2-3Galβ1-3)GαlNacα-Sp14	7	7
529	GlcNAcβ1-2 Manα1-6(GlcNAcβ1-4)(GlcNAcβ1-2Manα1-3)Manβ1-4GlcNAcβ1-4(Fucα1-6)GlcNAc-Sp21	7	4
134	GlcNAcβ1-6(Galβ1-3)GαlNacα-Sp14	7	10
295	Neu5Acα2-3Galβ1-4GlcNAcβ1-3Galβ1-3GlcNAcβ-Sp0	7	4
300	Galβ1-3Galβ1-4GlcNAcβ-Sp8	7	4
390	Galβ1-3GlcNAcβ1-3GαlNacα-Sp14	7	6
593	GlcNAcβ1-3Galβ1-4GlcNAcβ1-6(Galβ1-3)GαlNacα-Sp14	7	1
597	GlcNAcβ1-3Galβ1-4GlcNAcβ1-3Galβ1-4GlcNAcβ1-3GαlNacα-Sp14	6	3
144	Galβ1-3GalNAcβ1-4(Neu5Acα2-3)Galβ1-4Glcβ-Sp0	6	7
256	Neu5Acα2-3Galβ1-4(Fucα1-3)GlcNAcβ1-3Galβ-Sp8	6	3
305	Galβ1-4GlcNAcβ1-6Galβ1-4GlcNAcβ-Sp0	6	3
337	GlcNAca1-4Galβ1-4GlcNAcβ1-3Galβ1-4GlcNAcβ1-3Galβ1-4GlcNAcβ-Sp0	6	2
376	Neu5Acα2-6Galβ1-4GlcNAcβ1-3GalNAc-Sp14	6	5
431	GlcNAcβ1-2Manα1-6(GlcNAcβ1-4)(GlcNAcβ1-4(GlcNAcβ1-2)Manα1-3)Manβ1-4GlcNAcβ1-4GlcNAc-Sp21	6	3
513	GalNAcβ1-4(6S)GlcNAc-Sp8	6	5
568	(3S)GlcAβ1-3Galβ1-4GlcNAcβ1-3Galβ1-4Glc-Sp0	6	2
340	GlcNAca1-4Galβ1-4GlcNAcβ1-3Galβ1-4Glcβ-Sp0	6	4
205	KDNa2-3Galβ1-3GlcNAcβ-Sp0	6	5
227	Neu5Acα2-8Neu5Acα2-8Neu5Acα2-3Galβ1-4Glcβ-Sp0	6	3
428	Galβ1-4GlcNAcβ1-6(Fucα1-2Galβ1-3GlcNAcβ1-3)Galβ1-4Glc-Sp21	6	2
450	GαlNacα1-3(Fucα1-2)Galβ1-4GlcNAcβ1-6(GαlNacα1-3(Fucα1-2)Galβ1-4GlcNAcβ1-3)GalNAc-Sp14	6	1
517	(6P)Galβ1-4GlcNAcβ-SP0	6	7
223	Neu5Acα2-3Galβ1-3GαlNacα-Sp8	5	6
285	Neu5Gca2-6GαlNacα-Sp0	5	4
432	GlcNAcβ1-6(GlcNAcβ1-2)Manα1-6(GlcNAcβ1-4)(GlcNAcβ1-2Manα1-3)Manβ1-4GlcNAcβ1-4GlcNAc-Sp21	5	2
433	GlcNAcβ1-6(GlcNAcβ1-2)Manα1-6(GlcNAcβ1-4)(GlcNAcβ1-4(GlcNAcβ1-2)Manα1-3)Manβ1-4GlcNAcβ1-4GlcNAc-Sp21	5	2
251	Neu5Acα2-3Galβ1-4(6S)GlcNAcβ-Sp8	5	4
276	Neu5Acα2-8Neu5Acα2-3Galβ1-4Glcβ-Sp0	5	2
459	Neu5Acα2-3Galβ1-4GlcNAcβ1-4Manα1-6(GlcNAcβ1-4)(Neu5Acα2-3Galβ1-4GlcNAcβ1-4(Neu5Acα2-3Galβ1-4GlcNAcβ1-2)Manα1-3)Manβ1-4GlcNAcβ1-4GlcNAcβ-Sp21	5	1

109	Galα1-4(Galα1-3)Galβ1-4GlcNAcβ-Sp8	5	7
183	GlcNAcβ1-3Galβ1-4GlcNAcβ-Sp8	5	1
186	GlcNAcβ1-4-MDPLys	5	4
191	GlcNAcβ1-4GlcNAcβ1-4GlcNAcβ-Sp8	5	2
598	Galβ1-4GlcNAcβ1-3Galβ1-3GαlNacα-Sp14	5	2
396	GlcNAcβ1-2Manα1-6(Galβ1-4GlcNAcβ1-2Manα1-3)Manβ1-4GlcNAcβ1-4GlcNAc-Sp12	5	3
401	GαlNacα1-3GalNAcβ1-3Galα1-4Galβ1-4GlcNAcβ-Sp0	5	2
518	GαlNacα1-3(Fucα1-2)Galβ1-4GlcNAcβ1-6GalNAc-Sp14	5	3
304	Galβ1-4GlcNAca1-6Galβ1-4GlcNAcβ-Sp0	4	5
461	Neu5Acα2-3Galβ1-4GlcNAcβ1-6(Neu5Acα2-3Galβ1-4GlcNAcβ1-2)Manα1-6(GlcNAcβ1-4)(Neu5Acα2-3Galβ1-4GlcNAcβ1-4(Neu5Acα2-3Galβ1-4GlcNAcβ1-2)Manα1-3)Manβ1-4GlcNAcβ1-4GlcNAcβ-Sp21	4	4
174	GlcNAca1-6Galβ1-4GlcNAcβ-Sp8	4	2
184	GlcNAcβ1-3Galβ1-4GlcNAcβ1-3Galβ1-4GlcNAcβ-Sp0	4	3
412	Galα1-3(Fucα1-2)Galβ1-4GlcNAcβ1-3GαlNacα-Sp14	4	2
478	Neu5Acα2-3Galβ1-4GlcNAcβ1-6GαlNacα-Sp14	4	1
505	GαlNacα1-3(Fucα1-2)Galβ1-3GlcNAcβ1-6GαlNacα-Sp14	4	2
326	Galβ1-4(Fucα1-3)GlcNAcβ1-2Manα1-6(Galβ1-4(Fucα1-3)GlcNAcβ1-2Manα1-3)Manβ1-4GlcNAcβ1-4GlcNAcβ-Sp20	4	1
406	Galβ1-3GlcNAca1-6Galβ1-4GlcNAcβ-Sp0	4	1
440	Neu5Acα2-3Galβ1-4GlcNAcβ1-3Galβ-Sp8	4	2
163	Galβ1-4GlcNAcβ1-3Galβ1-4GlcNAcβ-Sp0	3	4
241	Neu5Acα2-3Galβ1-4(Neu5Acα2-3Galβ1-3)GlcNAcβ-Sp8	3	3
278	Neu5Acβ2-6GαlNacα-Sp8	3	3
392	GαlNacα1-3(Fucα1-2)Galβ1-3GαlNacα1-3(Fucα1-2)Galβ1-4GlcNAcβ-Sp0	3	3
538	GlcNAcβ1-3Galβ1-4GlcNAcβ1-2Manα1-6(GlcNAcβ1-3Galβ1-4GlcNAcβ1-2Manα1-3)Manβ1-4GlcNAcβ1-4GlcNAcβ-Sp12	3	6
554	Neu5Gca2-8Neu5Gca2-3Galβ1-4GlcNAcβ1-3Galβ1-4GlcNAc-Sp0	3	1
17	GlcNAcβ-Sp8	3	6
308	Neu5Acα2-6Galβ1-4GlcNAcβ1-2Manα1-6(GlcNAcβ1-2Manα1-3)Manβ1-4GlcNAcβ1-4GlcNAcβ-Sp12	3	1
363	Galβ1-4GlcNAcβ1-2Manα1-6(Manα1-3)Manβ1-4GlcNAcβ1-4GlcNAcβ-Sp12	3	3
552	Neu5Acα2-8Neu5Gca2-3Galβ1-4GlcNAc-Sp0	2	1
64	Fucα1-2Galβ1-3GalNAcβ1-4(Neu5Acα2-3)Galβ1-4Glcβ-Sp9	2	6
255	Neu5Acα2-3Galβ1-4(Fucα1-3)GlcNAcβ-Sp8	2	2
328	Neu5,9Ac2a2-3Galβ1-3GlcNAcβ-Sp0	2	1
334	GαlNacα1-3(Fucα1-2)Galβ1-4GlcNAcβ1-3Galβ1-4GlcNAcβ-Sp0	2	2
380	Galβ1-3GαlNacα1-3(Fucα1-2)Galβ1-4GlcNAc-Sp0	2	2
404	Galα1-3Galβ1-4GlcNAcβ1-3GαlNacα-Sp14	2	1
556	Neu5Acα2-8Neu5Acα2-3Galβ1-4GlcNAc-Sp0	2	1
135	Neu5Acα2-6(Galβ1-3)GαlNacα-Sp8	2	6
243	Neu5Acα2-6(Neu5Acα2-3Galβ1-3)GαlNacα-Sp8	2	1
178	GlcNAcβ1-6(GlcNAcβ1-3)Galβ1-4GlcNAcβ-Sp8	2	3
54	Galβ1-4GlcNAcβ1-2Manα1-6(Galβ1-4GlcNAcβ1-2Manα1-3)Manβ1-4GlcNAcβ1-4GlcNAcβ-Sp12	1	1
414	GαlNacα1-3GalNAcβ1-3Galα1-4Galβ1-4Glcβ-Sp0	1	2
511	(6S)GalNAcβ1-4GlcNAc-Sp8	1	2
155	Galβ1-4(6S)Glcβ-Sp0	-2	4
